# Ionic Solid Matrices Applied via Low-Temperature Evaporation Enable High-Resolution and Sensitive MALDI Imaging of Metabolites

**DOI:** 10.1021/jasms.6c00219

**Published:** 2026-09-03

**Authors:** Toufik Mahamdi, Nadia Moltó, Mireia Arasa, Roger Giné, Jordi Rofes, María García-Altares, Maria Vinaixa, Oscar Yanes

**Affiliations:** † 204165 Universitat Rovira i Virgili, Department of Electronic Engineering, Tarragona 43007, Spain; ‡ CIBER de Diabetes y Enfermedades Metabólicas Asociadas (CIBERDEM), Instituto de Salud Carlos III, Madrid 28029, Spain; § Metabolomics Platform, Institut de Recerca Biomèdica Catalunya Sud, Hospital Universitari Sant Joan de Reus, Reus 43204, Spain

**Keywords:** spatial metabolomics, mass spectrometry Imaging, MALDI, ionic matrix

## Abstract

Matrix-Assisted Laser Desorption/Ionization Mass Spectrometry Imaging (MALDI-MSI) enables spatially resolved molecular analysis of biological tissues. However, imaging low-molecular-weight metabolites remains challenging because conventional organic matrices generate intense background signals in the low *m*/*z* region. Ionic matrices can reduce matrix-related interferences, improve spectral reproducibility, and enhance sensitivity. However, their application in MALDI-MSI has remained limited, largely due to the need for additional matrix preparation and the challenging wet deposition of viscous ionic liquid matrices, and has mainly focused on lipid imaging above *m*/*z* 500. Here, we present a novel approach combining ionic solid matrices (ISMs) with low-temperature thermal evaporation (LTE) to enhance MALDI-MSI performance for small metabolites. ISMs based on α-cyano-4-hydroxycinnamic acid (CHCA) and 2,5-dihydroxybenzoic acid (DHB) provided superior sensitivity and reduced matrix background. In particular, CHCA + *N*,*N*-diethylaniline enabled the detection and annotation of 69 metabolites with *m*/*z* < 500 in mouse brain tissue at 20 μm spatial resolution. When applied to mouse pancreas, this ISM allowed the first specific localization of melatonin and 3-iodotyrosine within the islets of Langerhans, opening new avenues for spatial metabolomics in endocrine research.

## Graphical abstract

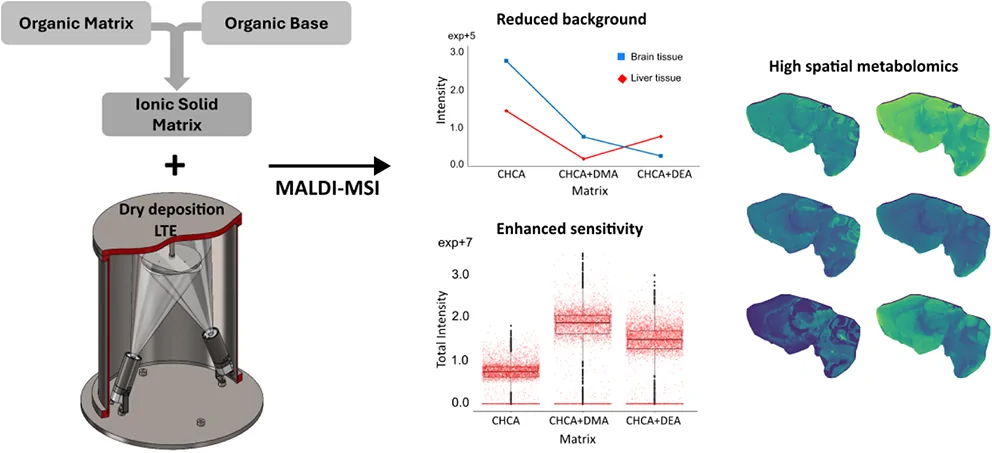

## 1 Introduction

Spatial metabolomics, enabled by Matrix-Assisted Laser Desorption/Ionization Mass Spectrometry Imaging (MALDI-MSI), has become a transformative technology for detecting and mapping the spatial distribution of metabolites, lipids, and other small molecules within biological tissues.^1^ By preserving the spatial context of molecular information, often at cellular or even subcellular resolution,^2^ MALDI-MSI offers unique insights into tissue heterogeneity, biochemical processes, and disease mechanisms, playing a key role in biomarker discovery and spatially resolved molecular phenotyping.^3^

A key component of MALDI-MSI is the matrix compound, which is cocrystallized with the analyte on the tissue surface. The matrix absorbs laser energy and mediates the desorption and ionization of analyte molecules, playing a critical role in determining ionization efficiency, sensitivity, reproducibility, and the overall range of detectable metabolites.^4–8^ However, conventional organic matrices, such as those based on cinnamic acids (e.g., α-cyano-4-hydroxycinnamic acid, CHCA) and benzoic acids (e.g., 2,5-dihydroxybenzoic acid, DHB), generate substantial background signals in the low-mass range, typically below *m*/*z* 500. These matrix-derived ions can interfere with the ionization, detection, and accurate quantification of small molecules.^9^

To address these limitations and improve MALDI-MSI performance for metabolite analysis, a range of strategies have been developed. These include the incorporation of matrix additives,^9^ the design of novel matrices with tailored physicochemical properties,^10–13^ the use of high molecular weight matrices to reduce background interference, and on-tissue derivatization protocols that chemically shift analytes to higher *m*/*z* values.^14–16^ Additional approaches involve the use of metal nanoparticles^17^ and optimized matrix deposition techniques,^18–20^ all aimed at enhancing ionization efficiency and signal-to-noise ratio for small molecules detection.

In this context, ionic matrices^21^ have also emerged as a promising alternative to conventional organic matrices. Ionic matrices, available in either liquid (ionic liquid matrices, ILMs) or solid (ionic solid matrices, ISMs) forms, are organic salts formed through acid–base reactions, typically involving equimolar mixtures of standard acidic MALDI matrices (e.g., DHB or CHCA) and organic bases such as amines.^22,23^ Ionic matrices offer several advantages over conventional MALDI matrices. They exhibit greater thermal and vacuum stability and significantly reduce matrix-related background signals. These properties lead to cleaner mass spectra, improved reproducibility, and enhanced sensitivity for detecting small molecules, including both metabolites and lipids.^24^

Despite these benefits, the application of ionic matrices in MALDI-MSI remains limited.^25^ This is partly because ionic matrices are generally not commercially available and require additional preparation prior to use. Furthermore, the highly viscous nature of conventional ILMs complicates the formation of homogeneous coatings required for high-spatial-resolution MSI. So far, studies have primarily focused on ILMs, applied via wet deposition techniques such as spraying, and have largely been restricted to lipid imaging in the *m*/*z* > 500 range.^26–29^

To overcome these limitations, we combined ISMs with low-temperature thermal evaporation (LTE), a dry matrix deposition method that improves ionization efficiency, minimizes analyte diffusion, and enhances spatial resolution.^30^ We evaluated three ISMs: CHCA + *N*,*N*-diethylaniline (DEA), CHCA + *N*,*N*-dimethylaniline (DMA), and DHB + aniline (ANI), against conventional matrices CHCA and DHB in MALDI-MSI analyses of mouse brain, liver, and pancreas tissues. All ISMs demonstrated superior performance, enabling enhanced detection of low mass metabolites while substantially reducing matrix-related background signals. Notably, CHCA+DEA exhibited the most pronounced improvements, supporting high-resolution imaging of both brain and pancreatic tissues. This matrix facilitated the annotation of 69 endogenous metabolites in mouse brain sections. Furthermore, its applicability was further demonstrated in mouse pancreas, where it enabled the specific localization of several metabolites including, melatonin and 3-iodotyrosine within the islets of Langerhans.

## 2 Materials and Methods

### 2.1 Reagents and Chemicals

Ethanol (HPLC grade) was obtained from Scharlab S.L. The following compounds were purchased from Sigma-Aldrich: α-cyano-4-hydroxycinnamic acid (CHCA), 2,5-dihydroxybenzoic acid (DHB), aniline (ANI), *N*,*N*-diethylaniline (DEA), and *N*,*N*-dimethylaniline (DMA). Analytical standards of alanine, creatine, cholesterol, glutamic acid, glutamine, glutathione, 4-hydroxybenzoic acid (4-HB), 3-indoleacetic acid (3-IAA), 3-iodotyrosine, melatonin, 3-methylglutaryl carnitine, kynurenic acid (KYNA), taurine, and tyrosine were also purchased from Sigma-Aldrich. Microscope slides were obtained from Epredia, and conductive indium tin oxide (ITO)-coated slides were sourced from Bruker.

### 2.2 Tissue Sampling

Brain, liver, and pancreas tissues were harvested from C57BL/6 healthy mice, flash-frozen on dry ice, and stored at −80 °C until further processing. Tissue sections were prepared using a CryoStar NX50 cryostat (Thermo Fisher Scientific) at −20 °C, sectioned at a thickness of 10 μm. Sections were mounted onto ITO-coated microscope slides and immediately stored at −80 °C until analysis.

### 2.3 Synthesis of Ionic Solid Matrices

For the preparation of ionic matrices, a stock solution of the organic matrix was first prepared by dissolving 1 g of either DHB or CHCA in 50 mL of ethanol. An equimolar amount of the base (ANI, DEA, or DMA) was then added to the matrix solution to form the corresponding acid–base solution. The mixture was vortexed for 15 min to ensure homogeneity.

To obtain ionic solid matrices (ISMs), the solvent was removed by subjecting the solutions to a nitrogen gas stream using a solid-phase extraction setup. To expedite solvent evaporation, each acid–base solution was divided into four 50 mL glass bottles. After complete solvent removal, the resulting dried ISMs were collected into Eppendorf tubes for subsequent LTE deposition.

### 2.4 Matrix Application

Matrix coating was performed using a NanoPVD-T15A thermal evaporator (Moorfield Nanotechnology, United Kingdom), following the LTE protocol described by Mahamdi et al.^30^ A total of 300 mg of matrix material, either conventional or ionic solid matrices, were placed into the crucible of the LTE source. Tissue section slides were first dried in a desiccator and then mounted on the LTE substrate holder for matrix deposition.

After evacuating the chamber to a base pressure of 5 × 10^–5^ mbar, the initial temperature was set to 60 °C. The system was allowed to stabilize, defined as the point at which the power supply unit dropped to zero. Subsequently, the temperature was gradually increased in 5 °C increments until the target evaporation temperature for the specific matrix was reached (Supplementary Table 1). Once at this temperature, the substrate shutter was opened, allowing the vaporized matrix to coat the tissue surface. Deposition thickness was precisely controlled and monitored throughout the process, reaching 15,000 Å for DHB and DHB-based ISMs, and 9,000 Å for CHCA and CHCA-based ISMs, in accordance with the optimized deposition thicknesses previously reported by Mahamdi et al.^30^

### 2.5 H&E Staining of Tissue Sections

A standard hematoxylin and eosin (H&E) staining protocol was applied to consecutive sections adjacent to those analyzed by MALDI-MSI. The staining procedure was as follows: tissue sections were stained with hematoxylin for 30 s, followed by sequential washes with tap water, 1% acetic acid, and distilled water for 10 s each. The sections were then stained with eosin for 20 s and dehydrated through graded ethanol solutions (70%, 96%, and 100%) for 10 s each. Finally, the tissues were cleared in Neo-Clear for 40 s.

The stained sections were mounted with coverslips using Cytoseal XYL (Richard-Allan Scientific, Kalamazoo, MI, USA) and allowed to dry at room temperature. Histological images were acquired at 40× magnification using the Ocus40 microscope slide scanner (Grundium, Finland).

### 2.6 MALDI-MSI and MALDI-MS/MS Acquisition

MALDI-MSI and MS/MS analyses were performed using a dual-ion funnel MALDI/ESI injector (Spectroglyph, Kennewick, WA) coupled to an Orbitrap Exploris 120 mass spectrometer (Thermo Fisher Scientific, Bremen, Germany). For MSI acquisitions, spatial step sizes of 20 μm and 30 μm were used. Spectra were acquired over the *m*/*z* range 50–600 Da at a resolving power of 60,000 (fwhm at *m*/*z* 200).

Each matrix was applied to a single tissue section from each tissue type (brain and liver). The experimental design focused on comparing matrix performance across chemically distinct tissues rather than performing technical replicate analyses of serial tissue sections.

Raw MSI data were processed using rMSI2 R package (https://github.com/prafols/rMSI2),^31^ including spectral smoothing, peak picking, peak binning, and *m*/*z* alignment prior to ion image generation. Ion annotations were performed using rMSIannotation^32^ and tentatively identified by matching against the Human Metabolome Database (HMDB) (https://www.hmdb.ca/), considering the following adducts: [M+H]^+^, [M–H_2_O+H]^+^, [M+Na]^+^, [M+K]^+^, [M+2Na–H]^+^, and [M+2K–H]^+^, with a mass tolerance of 5 ppm. For MS/MS experiments, collision-induced dissociation (CID) was performed with a normalized collision energy of 30%.

For metabolites with available authentic standards, MS1 adducts and, where signal intensity permitted, isotopic patterns were compared between tissue and standard spectra. For selected metabolites, MS/MS spectra acquired from authentic standards were also used to support metabolite annotation.

## 3 Results and Discussion

### 3.1 Evaluation of Ionic Solid Matrices for MALDI-MSI

Six ionic matrices were synthesized by combining two conventional acidic MALDI matrices, CHCA and DHB, with three different organic bases: aniline (ANI), *N*,*N*-diethylaniline (DEA), and N,N-dimethylaniline (DMA). This resulted in six ionic matrix formulations: CHCA+ANI, CHCA+DEA, CHCA+DMA, DHB+ANI, DHB+DEA, and DHB+DMA. However, DHB+DEA and DHB+DMA did not yield solid products upon solvent evaporation and were therefore excluded from further analysis (Supplementary Table 1).

We next evaluated the suitability of the ISMs for deposition via low-temperature thermal evaporation (LTE). The deposition temperature for each ISM was optimized individually (Supplementary Table 1). Compared to their conventional counterparts, CHCA (100 °C) and DHB (80 °C), the ISMs required evaporation temperatures approximately 10–15 °C higher. This increase is likely due to the incorporation of base components, which exhibit lower volatility and therefore require higher temperatures for efficient evaporation.

Given that the acidic and basic components in ISMs have different volatility profiles, we assessed whether the optimized LTE conditions were sufficient to co-deposit both components onto substrate surfaces. To do so, we performed MALDI-MSI analyses of the matrix only area, confirming the presence of the protonated and sodium-adduct ions of the base molecules (ANI, DEA, DMA) of all ISM formulations (Supplementary Figure 1). These results confirm that both components were successfully deposited and that the resulting matrix coatings retained the characteristic properties of ionic matrices.

Next, we assessed the performance of ISMs deposited via LTE for the ionization and detection of small metabolites within the *m*/*z* 50–600 Da range. To compare the overall ionization efficiency of the different matrices, we first evaluated the total ion count (TIC) per pixel across all tissue samples. In both brain and liver tissues, ISMs consistently outperformed conventional matrices, producing higher TIC values. Among the CHCA-based ISMs, CHCA+DEA generated the highest TIC in brain tissue (Figure 1a), while CHCA+DMA yielded the strongest signal in liver samples (Figure 1b).

**Figure 1:**
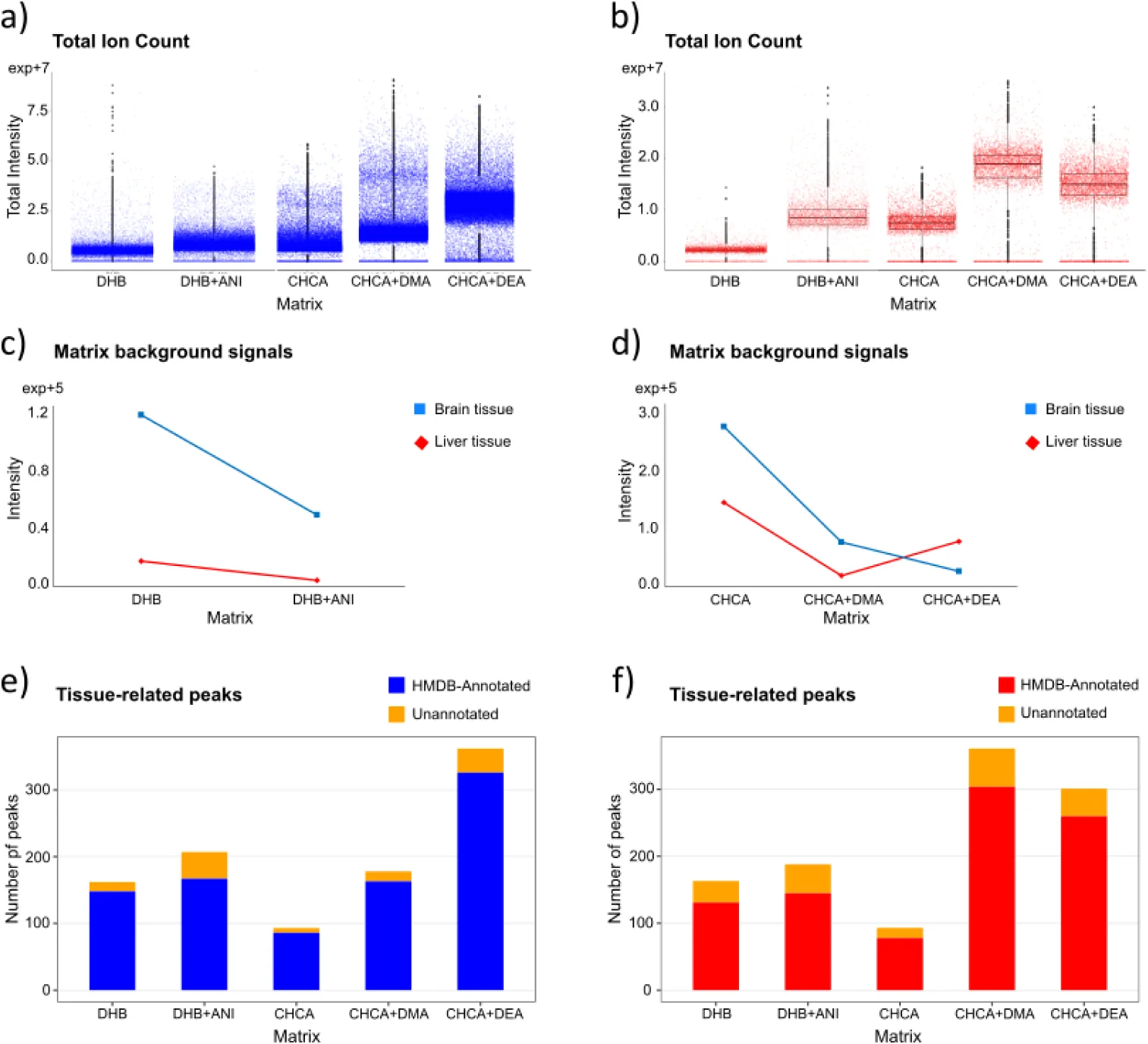
Comparative MALDI-MSI performance of DHB, DHB+ANI, CHCA, CHCA+DMA, and CHCA+DEA matrices. (a, b) boxplots of total ion count (TIC) intensities from mouse brain (a) and liver (b) tissue. (c, d) sum intensities of five characteristic matrix-related ions for DHB (c) and CHCA (d) detected in brain (blue) and liver (red) tissues. (e, f) Bar plots showing the number of tissue-related peaks after exclusion of matrix-related ions. Blue (brain) and red (liver) bars represent tissue-related features with a tentative HMDB annotation, whereas orange bars indicate tissue-related features without an HMDB match.

To investigate the source of these increased TIC values, we first examined the formation of the predominant matrix-related ion clusters associated with each ISM. For DHB and DHB-based ISMs, key signals included *m*/*z* 137.0239 ([M–H_2_O+H]^+^), *m*/*z* 155.0345 ([M+H]^+^), *m*/*z* 273.0399 ([2M–2H_2_O+H]^+^), and *m*/*z* 409.0559 ([3M–3H_2_O+H]^+^) (Supplementary Figure 2). For CHCA and CHCA-based ISMs, the main matrix-related ions were *m*/*z* 146.0613 ([M–CO+H]^+^), *m*/*z* 172.0394 ([M–H_2_O+H]^+^), *m*/*z* 190.0511 ([M+H]^+^), *m*/*z* 212.0340 ([M+Na]^+^), and *m*/*z* 228.0067 ([M+K]^+^) (Supplementary Figure 3). In all cases, regardless of tissue type, the signal intensity of matrix-related ions was significantly reduced in samples prepared with ISMs, resulting in lower overall matrix background (Figure 1c, d). These results strongly suggested that the enhanced TIC observed with ISMs was primarily due to improved ionization of endogenous metabolites rather than contributions from matrix-related ions. To confirm this, we quantified the number of biologically relevant signals detected in each condition. By comparing spectra acquired from tissue regions with those from adjacent matrix-only areas, we filtered out matrix-derived peaks and retained only tissue-specific ions. These were then matched against the Human Metabolome Database (HMDB) using a defined set of matrix-specific adducts (see Materials and Methods section for details).

Using the conventional DHB matrix, we annotated 148 and 131 endogenous monoisotopic peaks in brain and liver tissue, respectively. In contrast, the ISM DHB+ANI increased the number of annotated ions to 167 in the brain and 145 in liver samples (Figure 1e, f). The CHCA matrix performed less effectively, yielding only 86 and 78 annotated monoisotopic peaks in the brain and liver, respectively. However, when used as a ISM, CHCA+DMA and CHCA+DEA dramatically improved metabolite coverage, reaching 163 and 326 annotated ions in the brain, and 304 and 260 in liver tissue, respectively (Figure 1e, f). These results highlight the significant analytical advantage of combining ISMs with LTE, particularly for low molecular weight metabolite detection, within the *m*/*z* 50–600 Da range.

Given the substantial improvement in metabolite coverage achieved with the ISM CHCA+DEA, we focused our next analyses on brain tissue to validate these findings through high-confidence metabolite identification. Following annotation against the Human Metabolome Database (HMDB) with a 5 ppm mass tolerance, we aimed for level 1 identification confidence by comparing both MS1 adducts and MS/MS fragmentation patterns with authentic standards.

For example, an ion at *m*/*z* 265.9626, tentatively assigned to the [M+2K–H]^+^ adduct of kynurenic acid (KYNA), a neuroactive metabolite of the kynurenine pathway, and an ion corresponding to the [M+2K–H]^+^ adduct of taurine were detected in both the authentic standards and mouse brain tissue spectra (Figure 2a, b). To rule out potential misassignment arising from the isomeric composition shared by KYNA and CHCA (both C_10_H_7_NO_3_), we verified that the ion at *m*/*z* 265.9626 exhibited a distinct spatial distribution within the cerebellum compared with CHCA-related ions (Supplementary Figure 4a). Furthermore, this ion was absent from matrix-only regions (Supplementary Figure 4b), supporting its tissue-specific origin.

**Figure 2:**
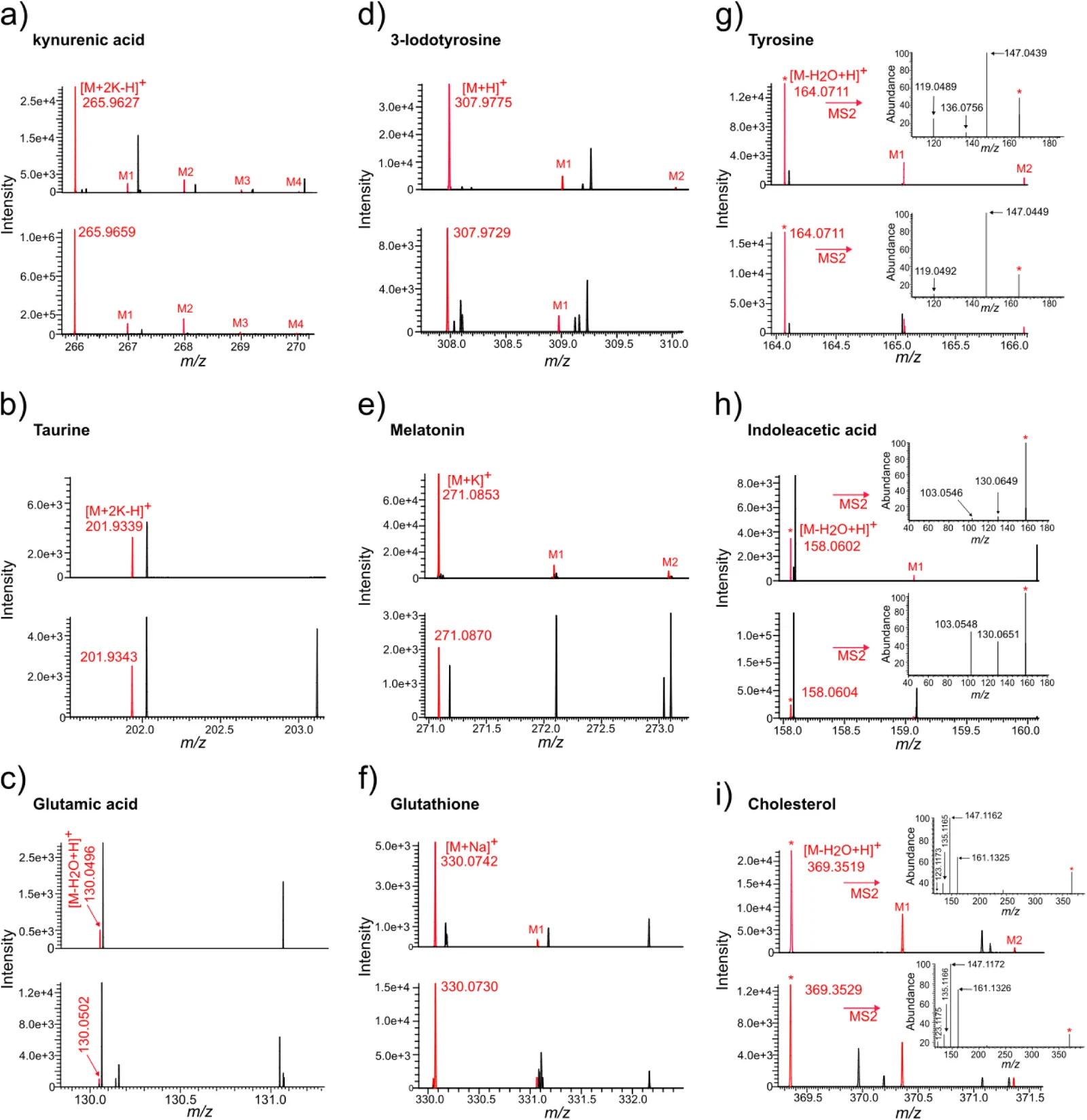
MS spectra of the selected LMWCs: (a) kynurenic acid, (b) taurine, (c) glutamic acid, (d) 3-iodotyrosine, (e) melatonin, and (f) glutathione. MS and MS/MS spectra of the selected LMWCs: (g) tyrosine, (h) indoleacetic acid, and (i) cholesterol. Spectra on top position were acquired from MALDI-MS analysis of standard compounds and those presented on bottom were obtained from MALDI-MSI of the mouse brain section, using the ISM CHCA+DEA. Peaks related to the selected compound, including isotopes, are colored in red. *: precursor ion for MS/MS analysis.

Further analysis revealed consistent adduct formation between standard and tissue samples for multiple metabolites. For instance, the dehydrated ion [M–H_2_O+H]^+^ of glutamic acid, the protonated ion [M+H]^+^ of 3-iodotyrosine (3IAA), the potassium adduct [M+K]^+^ of melatonin, and the sodium adduct [M+Na]^+^ of glutathione all showed reproducible signals in MS1 spectra (Figure 2c–f). Similar results were obtained for additional compounds, including 4-hydroxybenzoic acid (4-HB), alanine, creatine, glutamine, indoleacetic acid, and tyrosine, for which one or more adducts were confirmed across standard and tissue samples (Supplementary Figures 5–13). For ions with sufficient signal intensity, the observed isotopic patterns were compared with those of the corresponding standards, providing additional confidence in the putative annotations.

Moreover, high-quality MALDI-MS/MS spectra obtained directly from brain sections enabled structural confirmation of several metabolites. For tyrosine, 3IAA, and cholesterol, the fragmentation patterns closely matched those of the standards (Figure 2g–i). Additionally, MS/MS analysis allowed us to annotate fragments from endogenous compounds such as creatine, dopamine, spermine, *N*-methylserotonin, choline, and L-carnitine by comparison with reference spectra from HMDB entries, providing deeper insight into their fragmentation patterns (Supplementary Figure 14).

By combining the comparison of the observed MS1 adducts and isotopic patterns between authentic standards and tissue samples, and, where available, MS/MS spectral matching with authentic standards, we confidently annotated 69 metabolites below *m*/*z* 500 from brain tissue with the ISM CHCA+DEA (Supplementary Table 2). This included key neurotransmitters such as acetylcholine, dopamine, l-DOPA, and gamma-aminobutyric acid (GABA). Notably, only 14 of these 69 metabolites were also detected using conventional CHCA or DHB matrices, highlighting the enhanced ionization efficiency afforded by the ISM combined with LTE deposition.

In addition to enhanced molecular coverage, this method also provided significant improvements in spatial fidelity. In MALDI-MSI, particularly for small molecules and metabolites, spatial resolution is often compromised by nonuniform matrix application or the formation of large crystals, issues commonly associated with wet deposition methods. These factors can lead to analyte delocalization due to diffusion from their original locations. In contrast, LTE-deposited ISM produce uniform, submicrometer-sized crystals and allow precise control of matrix thickness, resulting in a high-purity, homogeneous matrix layer. A comparison of representative metabolite ion images acquired using the different ionic matrices is provided in Supplementary Figure S15. All tested ionic matrices produced high-quality MALDI-MSI images at 20 μm spatial resolution, confirming that LTE deposition is compatible with each matrix. However, the ISM CHCA+DEA consistently generated sharper ion images and enabled the detection of a larger number of metabolites, supporting its selection as the optimal matrix for subsequent analyses. As shown in Figure 3, MALDI-MSI of 15 selected metabolites at 20 μm resolution yielded sharp, well-defined ion images, demonstrating that the combination of ISMs and LTE enables high-resolution spatial metabolomic mapping of small molecules.

**Figure 3:**
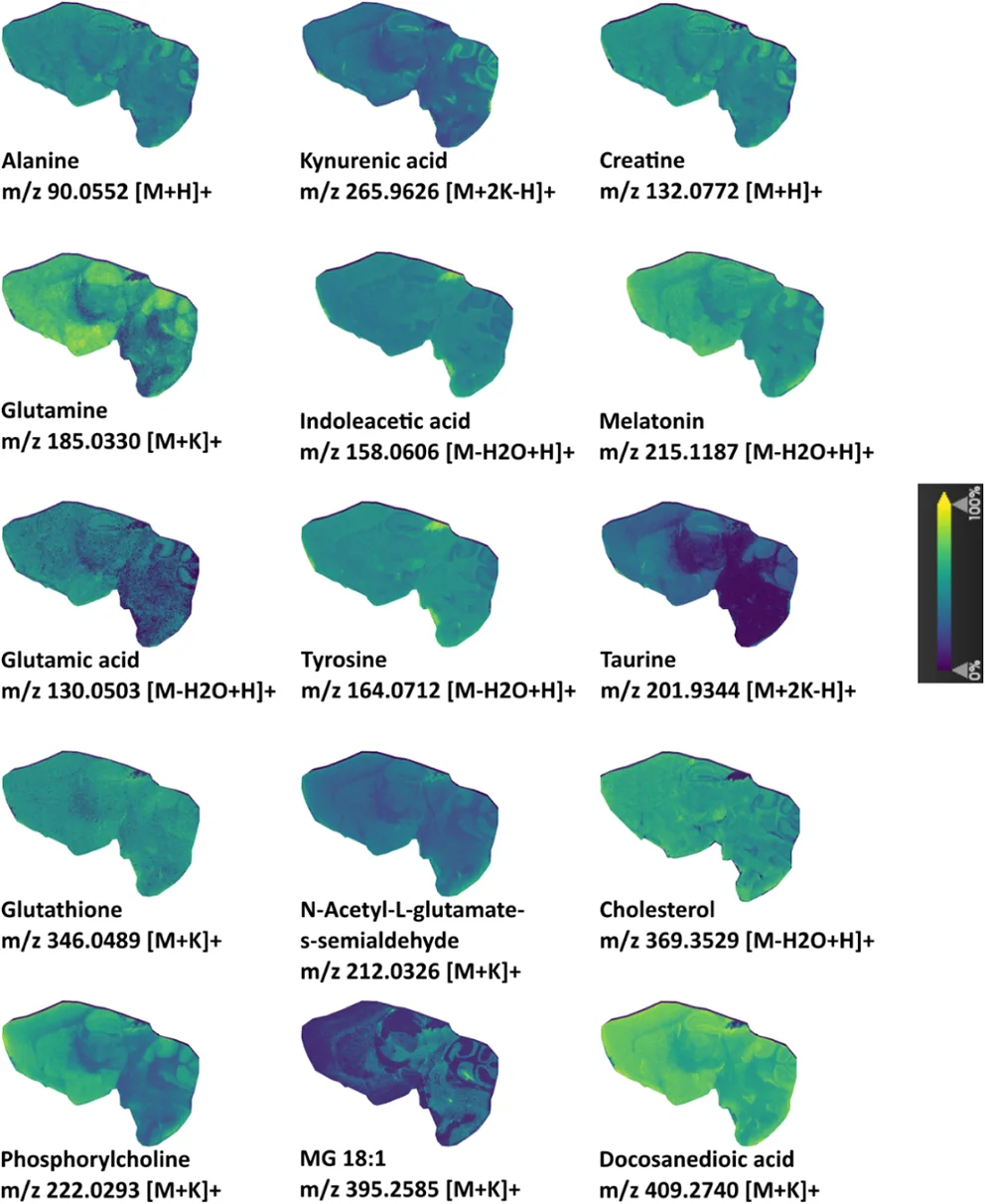
MALDI images of 15 selected metabolites in a mouse sagittal brain section, acquired at 20 μm spatial resolution using the ISM CHCA+DEA.

### 3.2 MALDI-MSI of Metabolites in Mouse Pancreatic Tissue

To further demonstrate the high-resolution and sensitive capabilities of MALDI-MSI for metabolite imaging, we applied the ISM CHCA+DEA to analyze the metabolic composition of the islets of Langerhans in the pancreas. Each islet consists of a central core of beta cells, accounting for up to 60% of islet mass, surrounded by a mantle of alpha cells (approximately 30%), along with smaller populations of delta, epsilon, and pancreatic polypeptide cells. Alpha cells secrete glucagon, the primary catabolic hormone that increases blood glucose and fatty acid levels, while beta cells produce insulin, which lowers blood glucose by promoting uptake into the liver, muscle, and adipose tissue.

Although MALDI-MSI has been widely used to image pancreatic peptide hormones and proteins,^33^ its application to lipids and small metabolites within islets has remained limited. Prior studies localized only a few lipid species, such as specific lysophosphatidylcholines (LPCs), phosphatidylcholines (PCs), and GM3 gangliosides, within murine islet cells,^34^ findings that were also supported by nano-DESI MSI.^35^ For small metabolites, compounds like glucose 6-phosphate, fatty acids, ADP, cholesterol sulfate, and 3-*O*-sulfogalactosylceramide have shown considerable intra- and interislet variability.^36^

Using the ISM CHCA+DEA, multiple small metabolites were annotated in the islets of Langerhans (Supplementary Table 3). Representative ion images acquired at 25 μm spatial resolution are shown in Figure 4, illustrating the distinct spatial localization of selected metabolites within the islets. These include ions at *m*/*z* 215.1185 and *m*/*z* 297.1266, identified as melatonin and phenylalanyl-methionine, respectively, were enriched along the periphery of the islets. Conversely, metabolites such as 3-methylglutarylcarnitine (*m*/*z* 213.1413), 3-iodotyrosine (*m*/*z* 307.9779), and 6-beta-hydroxycortisol (*m*/*z* 417.1726) were more uniformly distributed throughout the islets, with no significant difference between core and edge regions.

**Figure 4:**
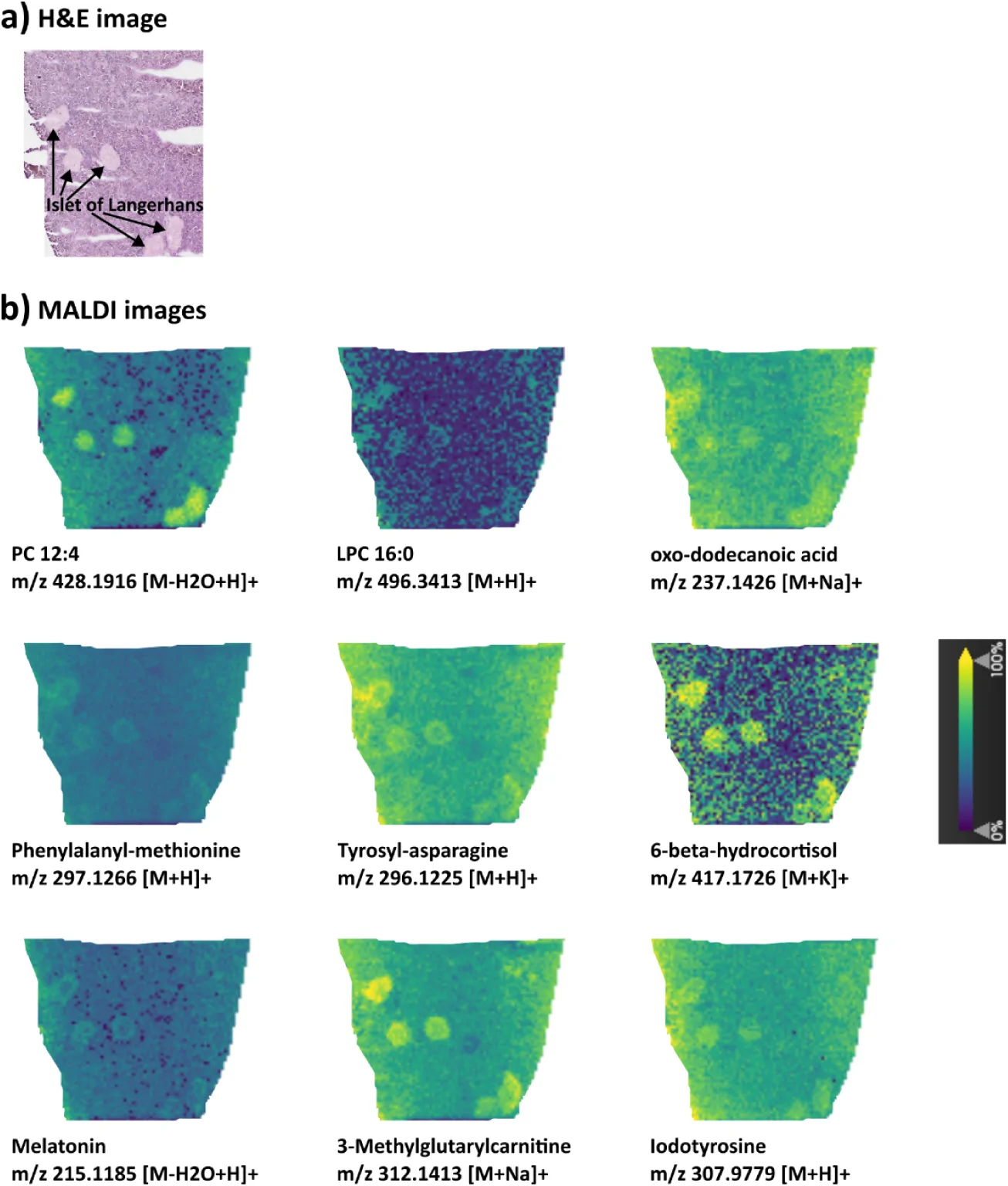
Spatial localization of small metabolites in mouse pancreas. (a) H&E-stained image of pancreas tissue with five islets of Langerhans indicated by black arrows. (b) MALDI images of nine selected metabolites acquired at 25 μm spatial resolution using the solid ionic matrix CHCA+DEA, showing specific localization to the islets of Langerhans.

Melatonin plays a key regulatory role in insulin and glucagon secretion through its interaction with MTNR1A and MTNR1B receptors, which are expressed in both α- and β-cells.^37^ Although MTNR1A protein expression has previously been localized to peripherally positioned β-cells, to our knowledge, this is the first report of direct detection of melatonin within pancreatic islets. This finding provides MALDI MSI as a useful and sensitive technique to potentially link circadian rhythms and metabolic homeostasis, highlighting melatonin’s dual role in modulating both insulin and glucagon output.

Iodotyrosine is a precursor in TH synthesis, and TH receptors (TRα/TRβ) are expressed in pancreatic islets.^38,39^ The presence of iodotyrosine implies local TH metabolism, which could fine-tune insulin release in response to nutrient availability.

## 4 Conclusions

We present a novel strategy for enhancing MALDI-MSI of low molecular weight compounds: the application of ionic solid matrices (ISMs) via low-temperature thermal evaporation (LTE). Compared to conventional matrices such as CHCA and DHB, ISMs offer clear advantages: (1) a significant reduction in matrix-related ion signals, minimizing spectral interference with analyte peaks, and (2) improved sensitivity and signal intensities for tissue-derived ions.

Using the ISM CHCA+DEA, we demonstrated reduced matrix cluster formation and enhanced ionization efficiency in mouse brain tissue, enabling the detection and annotation of 69 small metabolites (*m*/*z* < 500). The resulting MALDI-MSI data at 20 μm resolution provided high-quality images with clear spatial localization of metabolites within brain regions.

Finally, while MALDI-MSI has traditionally focused on imaging peptide hormones^40–42^ and lipids^34,35^ in the pancreas, application of CHCA+DEA with LTE enabled the detection and specific localization of metabolites, such as melatonin and 3-iodotyrosine, within the islets of Langerhans. These findings offer valuable insights into the pancreatic metabolic landscape and open new opportunities for advancing our understanding of islet biology, function, and disease.

## Supplementary Material



## Notes

Evaluation of synthesized ionic matrices, MALDI-MS spectra acquired from matrix only section, plot intensity of the key matrix related ions, representative MALDI images from mouse cerebellum of the selected matrix-derived ions, representative MALDI-MS spectra acquired from the dried-droplet metabolite standard analysis and brain section imaging, representative MALDI-MS/MS spectra acquired from brain section imaging using the ionic solid matrix CHCA+DEA, representative MALDI images of mouse brain acquired from the ionic solid matrices, list of LMWCs annotated from MALDI-MSI of mouse brain using the ionic solid matrix CHCA+DEA, representative MALDI-MS/MS spectra acquired from the pancreas section imaging using the ionic solid matrix CHCA+DEA, and list of LMWCs annotated from MALDI-MSI of mouse pancreatic islets using the ionic solid matrix CHCA+DEA (PDF)
