# Supplementary material for: Ionic Solid Matrices Applied via Low-Temperature Evaporation Enable High-Resolution and Sensitive MALDI Imaging of Metabolites
Source: J Am Soc Mass Spectrom. 2026 Sep 3;37(10):2463–71. doi: 10.1021/jasms.6c00219 (PMC13651598; doi:10.1021/jasms.6c00219)
Supplement: Supplementary file 1 [file js6c00219_si_001.pdf]

Ionic Solid Matrices applied via Low-Temperature Evaporation enable High-Resolution and Sensitive MALDI Imaging of Metabolites

Toufik Mahamdi[1,2], Nadia Moltó[1,2], Mireia Arasa[1], Roger Giné[1,2], Jordi Rofes[1,2], María García-Altares[1], Maria Vinaixa[1,2,3], Oscar Yanes[1,2,3]*

1. Universitat Rovira i Virgili, Department of Electronic Engineering, IISPV, 43007 Tarragona, Spain.

2. CIBER de Diabetes y Enfermedades Metabólicas Asociadas (CIBERDEM), Instituto de Salud Carlos III, Madrid, Spain.

3. Metabolomics Platform, Institut de Recerca Biomèdica Catalunya Sud, Hospital Universitari Sant Joan de Reus, 43204 Reus, Spain

* Corresponding author: oscar.yanes@urv.cat

Key words: Spatial metabolomics, Mass Spectrometry Imaging, MALDI, Ionic Matrix.

Table S1: Evaluation of ionic matrices, in terms of structure, physical state, and the optimised temperature for low-temperature thermal evaporation deposition

Matrix	Structure	State of aggregation (at 25ºC)	LTE temperature
CHCA	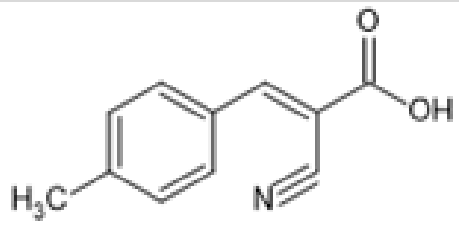	Solid	100 ºC
CHCA+ANI	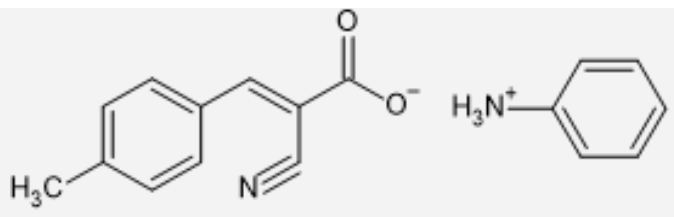	Solid	112 ºC
CHCA+DEA	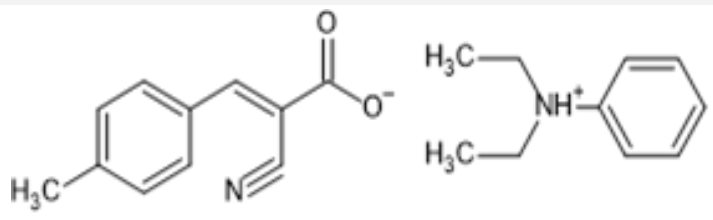	Solid	115 ºC
CHCA+DMA	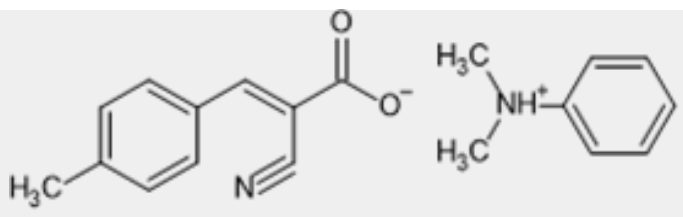	Solid	115 ºC
DHB	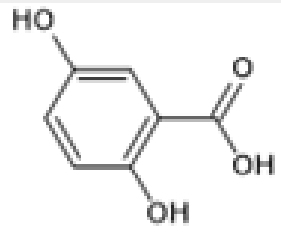	Solid	80 ºC
DHB+ANI	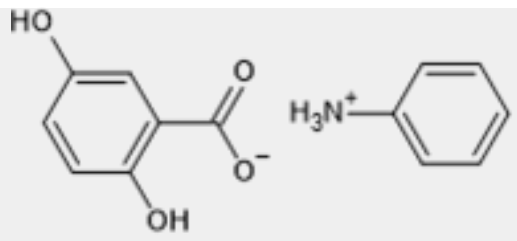	Solid	90 ºC
DHB+DEA	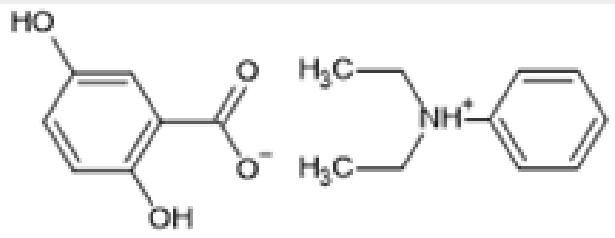	Liquid	/
DHB+DMA	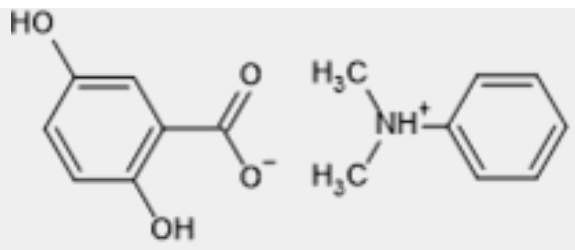	Liquid	/

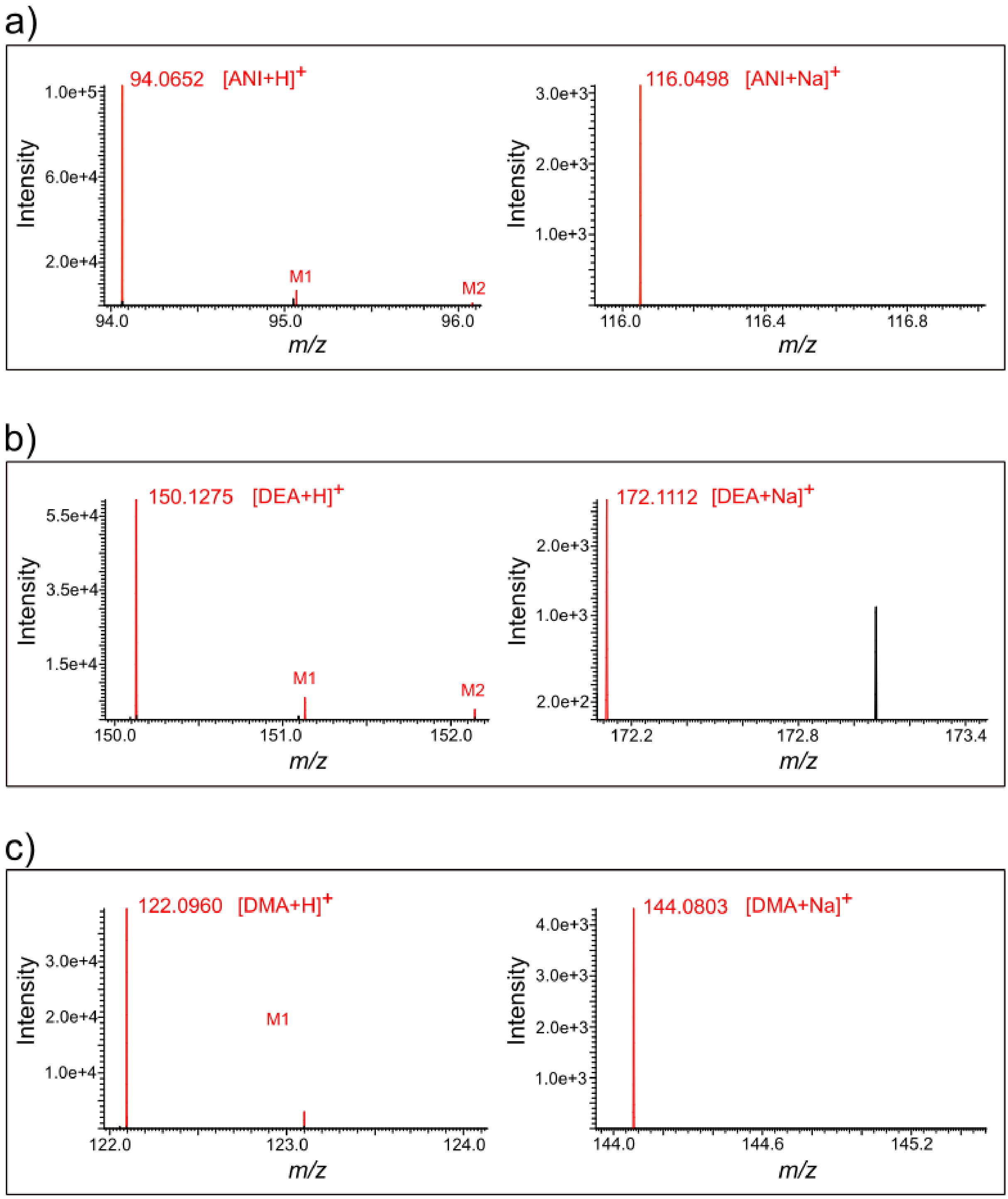

Figure S1: MALDI-MS spectra acquired from matrix only section showing the detection of peaks from the base components of the solid ionic matrix; (a) the protonated ion (*m/z* 94.0652) and the sodium adduct ion (*m/z* 116.0498) of aniline added to DHB matrix; (b) the protonated ion (*m/z* 150.1275) and the sodium adduct ion (*m/z* 172.1112) of N,N-diethylaniline added to CHCA matrix; (c) the protonated ion (*m/z* 122.0960) and the sodium adduct ion (*m/z* 144.0803) of N,N-dimethylaniline added to CHCA matrix.

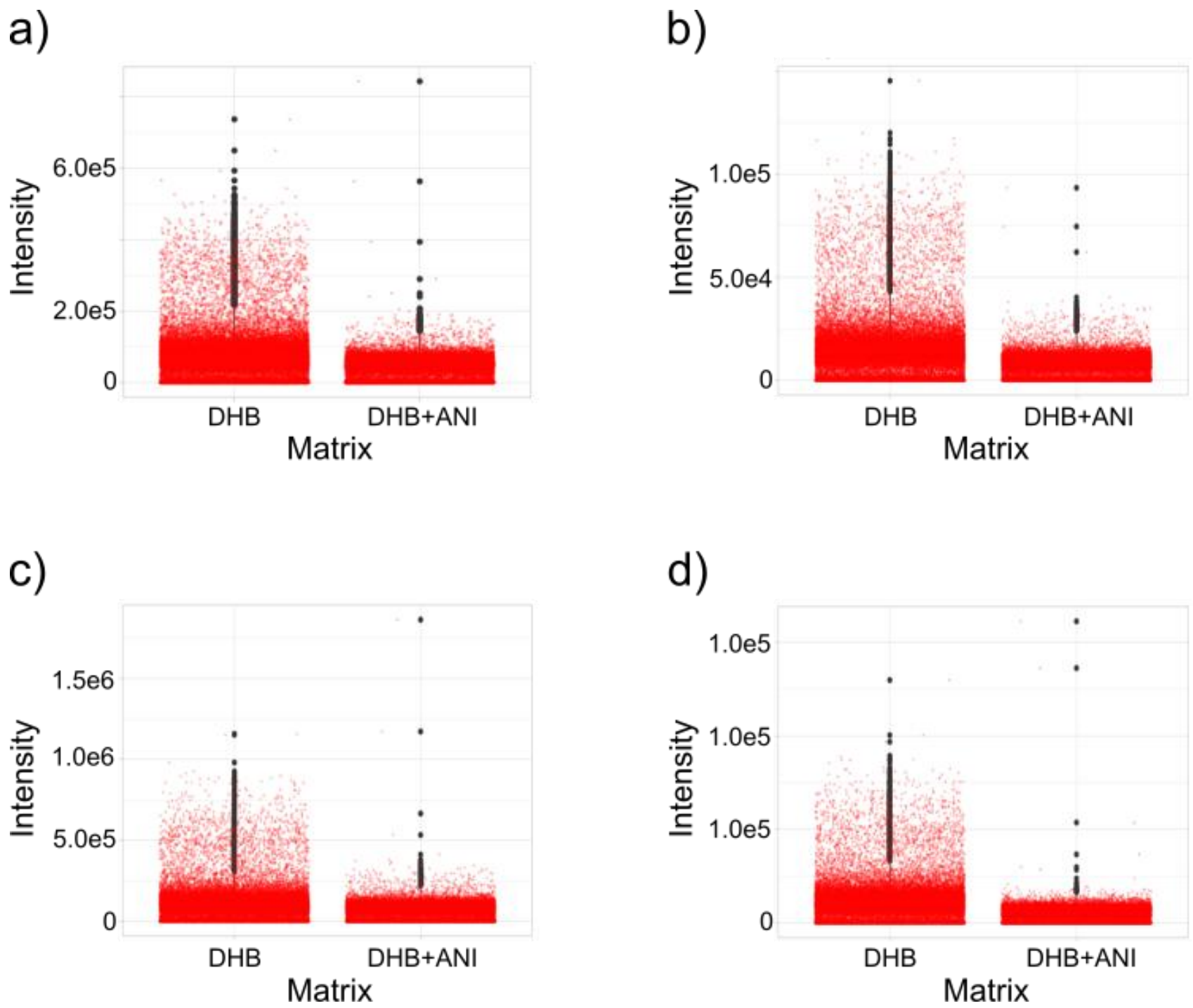

Figure S2: Plot intensity of the key DHB related matrix ions detected using DHB matrix and the solid ionic matrix DHB+ANI; (a) *m/z* 137.0239 ([M-H$_2$O+H]$^+$), (b) *m/z* 155.0345 ([M+H]$^+$), (c) *m/z* 273.0399 ([2M-2(H$_2$O)+H]$^+$), and (d) *m/z* 409.0559 ([3M-3(H$_2$O)+H]$^+$).

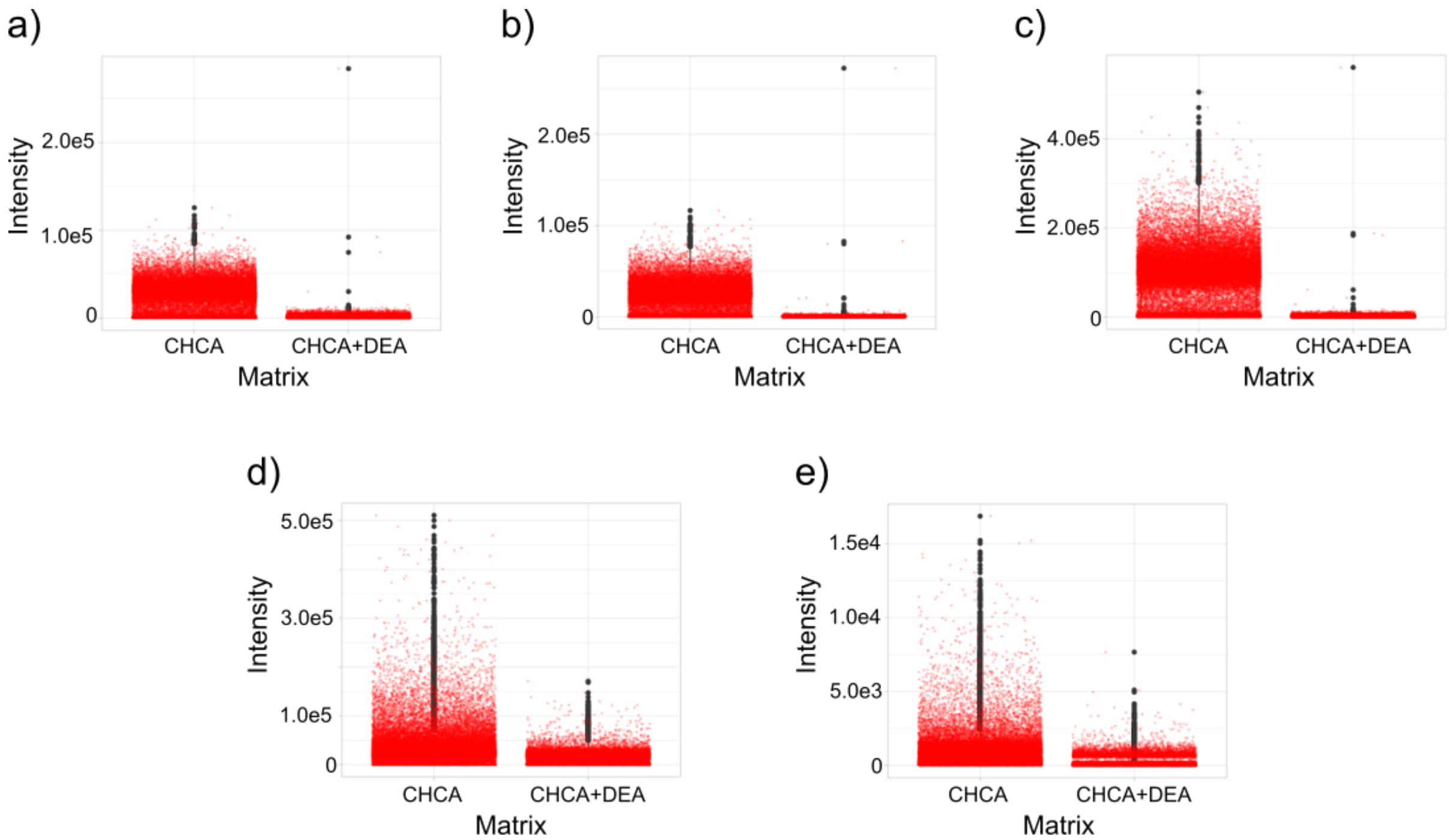

Figure S3: Plot intensity of the key CHCA related matrix ions detected using CHCA matrix and the solid ionic matrix CHCA+DEA; (a) *m/z* 146.0613 ([M-CO+H]$^+$), (b) *m/z* 172.0394 ([M-H2O+H]$^+$), (c) *m/z* 190.0511 ([M+H]$^+$), (d) *m/z* 212.0340 ([M+Na]$^+$), and (e) *m/z* 228.0067 ([M+K]$^+$).

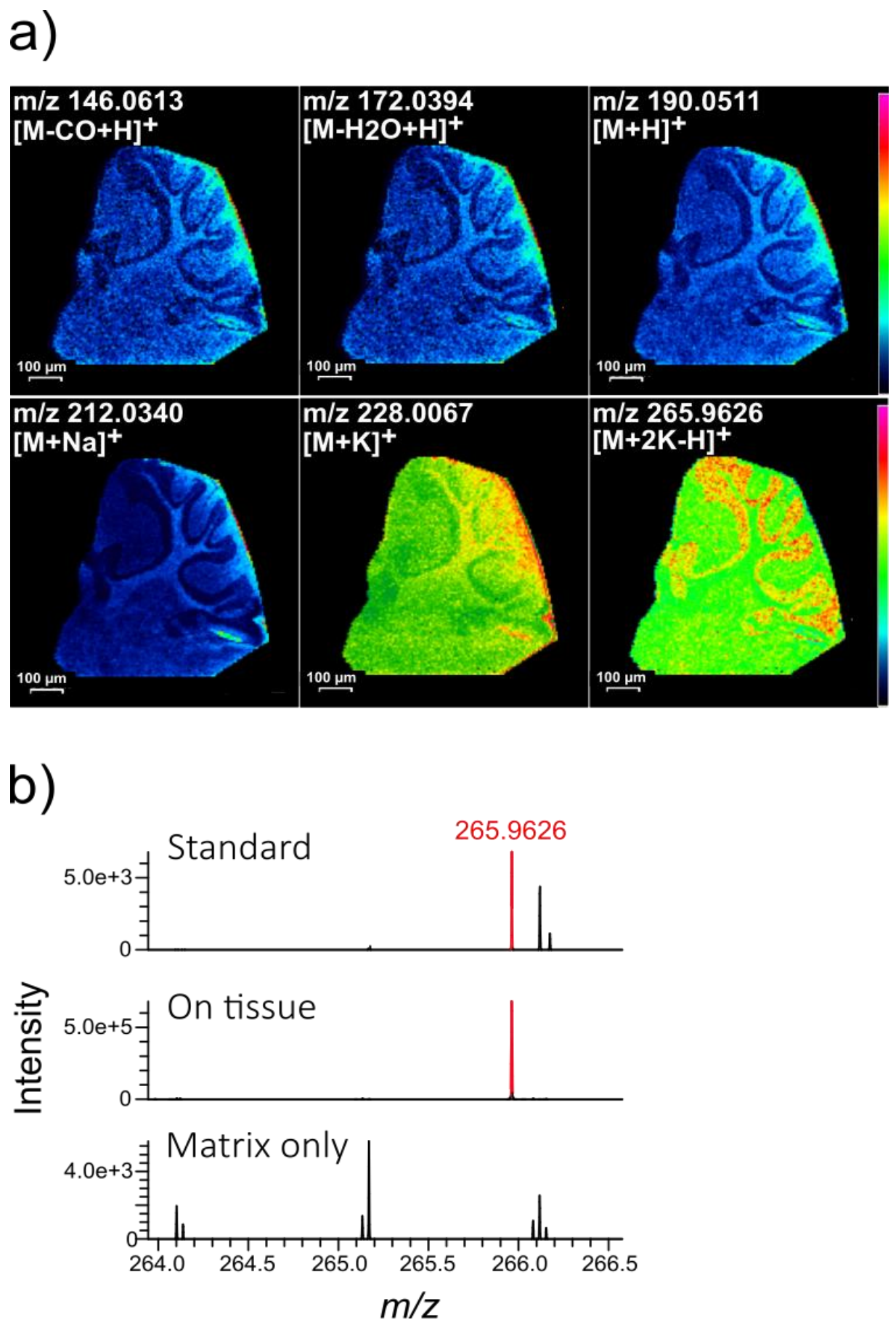

Figure S4: (a) MALDI images of mouse cerebellum acquired at 30 µm, using the solid ionic matrix CHCA+DEA, of the detected ions at *m/z* 146.0613, *m/z* 172.0394, *m/z* 190.0511, *m/z* 212.0340, *m/z* 228.0067, and *m/z* 265.9626 annotated as $[M\text{-}CO+H]^+$, $[M\text{-}H_2O+H]^+$, $[M+H]^+$, $[M+Na]^+$, $[M+K]^+$, and $[M+2K\text{-}H]^+$ respectively. (b) MLADI-MS spectra showing the detection the of the peak at *m/z* 265.9626 from the standard solution and from the brain tissue, but not from the matrix only measurements.

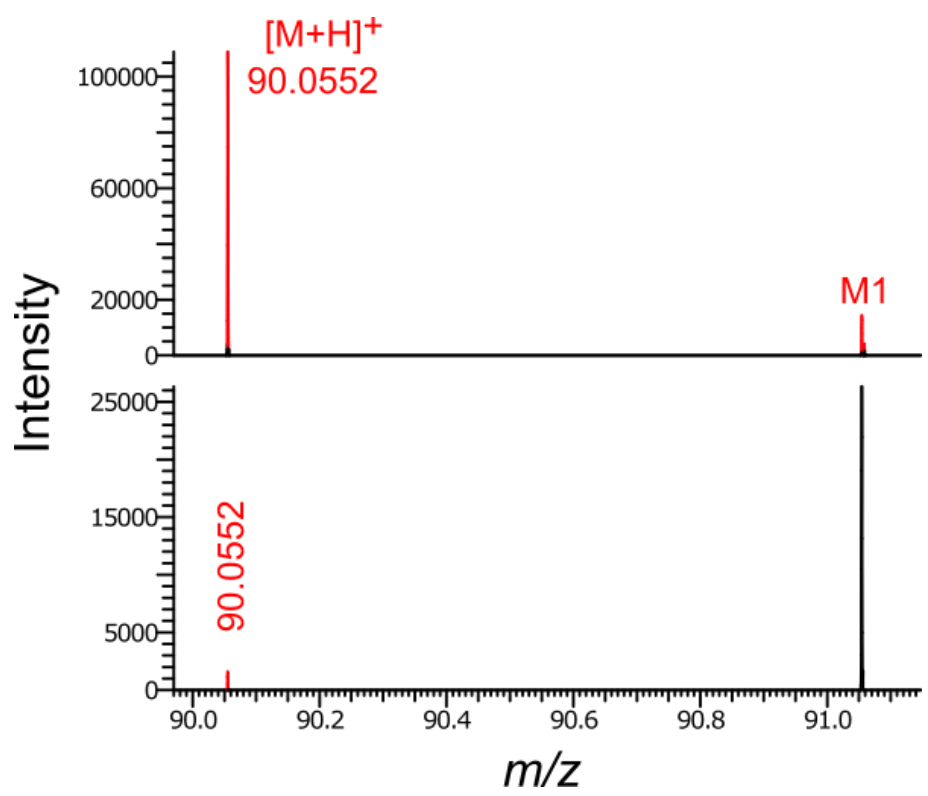

Figure S5: MALDI-MS spectra acquired from the dried-droplet of alanine standard analysis (top position) and brain section imaging (bottom position) using the ISM CHCA+DEA.

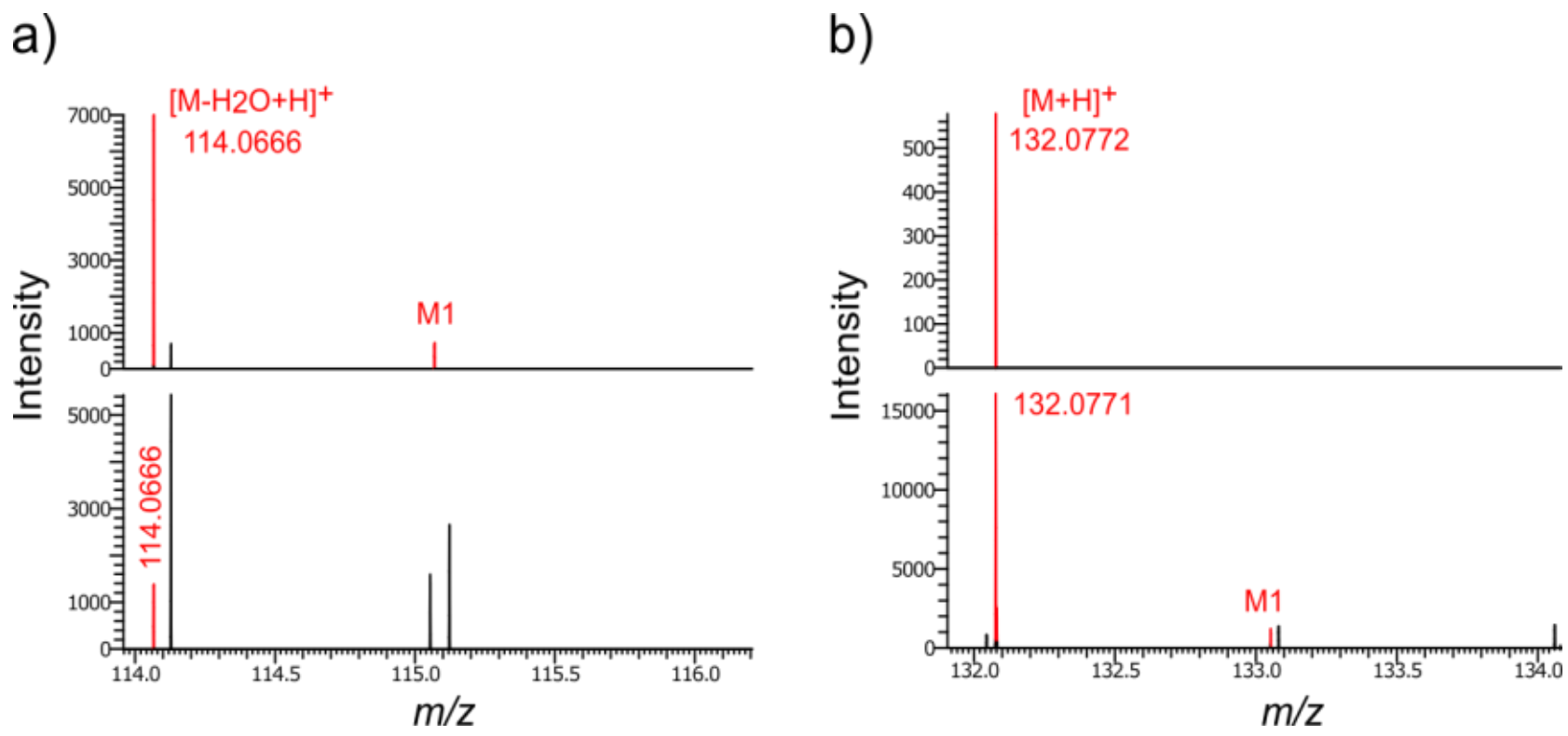

Figure S6: MALDI-MS spectra acquired from the dried-droplet of creatine standard analysis (top position) and brain section imaging (bottom position) using the ISM CHCA+DEA: (a) zoomed in at the peak *m/z* 114.06 ([M-H_2O+H]$^+$) and (b) zoomed in at the peak *m/z* 132.07 ([M+H]$^+$).

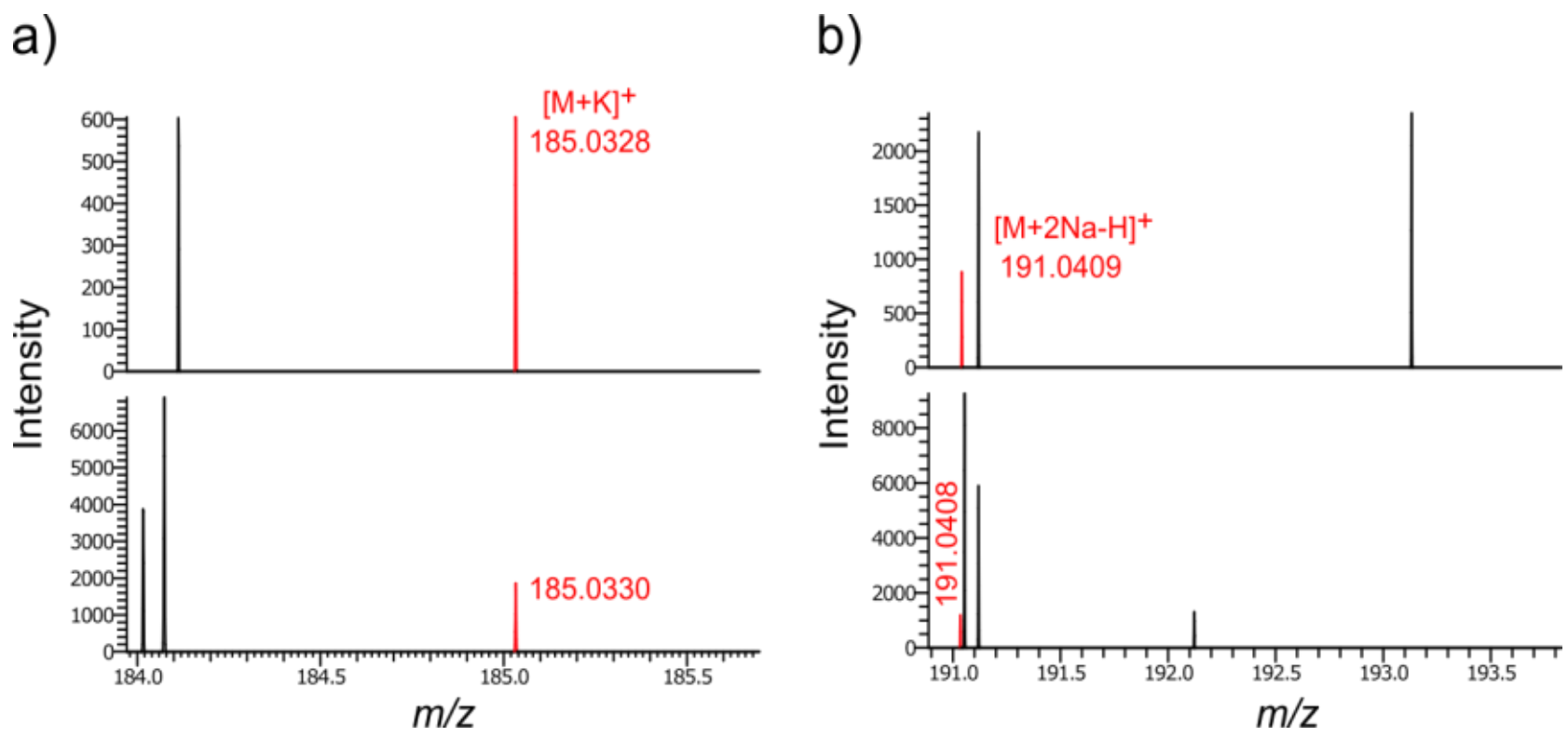

Figure S7: MALDI-MS spectra acquired from the dried-droplet of glutamine standard analysis (top position) and brain section imaging (bottom position) using the ISM CHCA+DEA: (a) zoomed in at the peak *m/z* 185.03 ([M+K]$^+$) and (b) zoomed in at the peak *m/z* 191.04 ([M+2Na-H]$^+$).

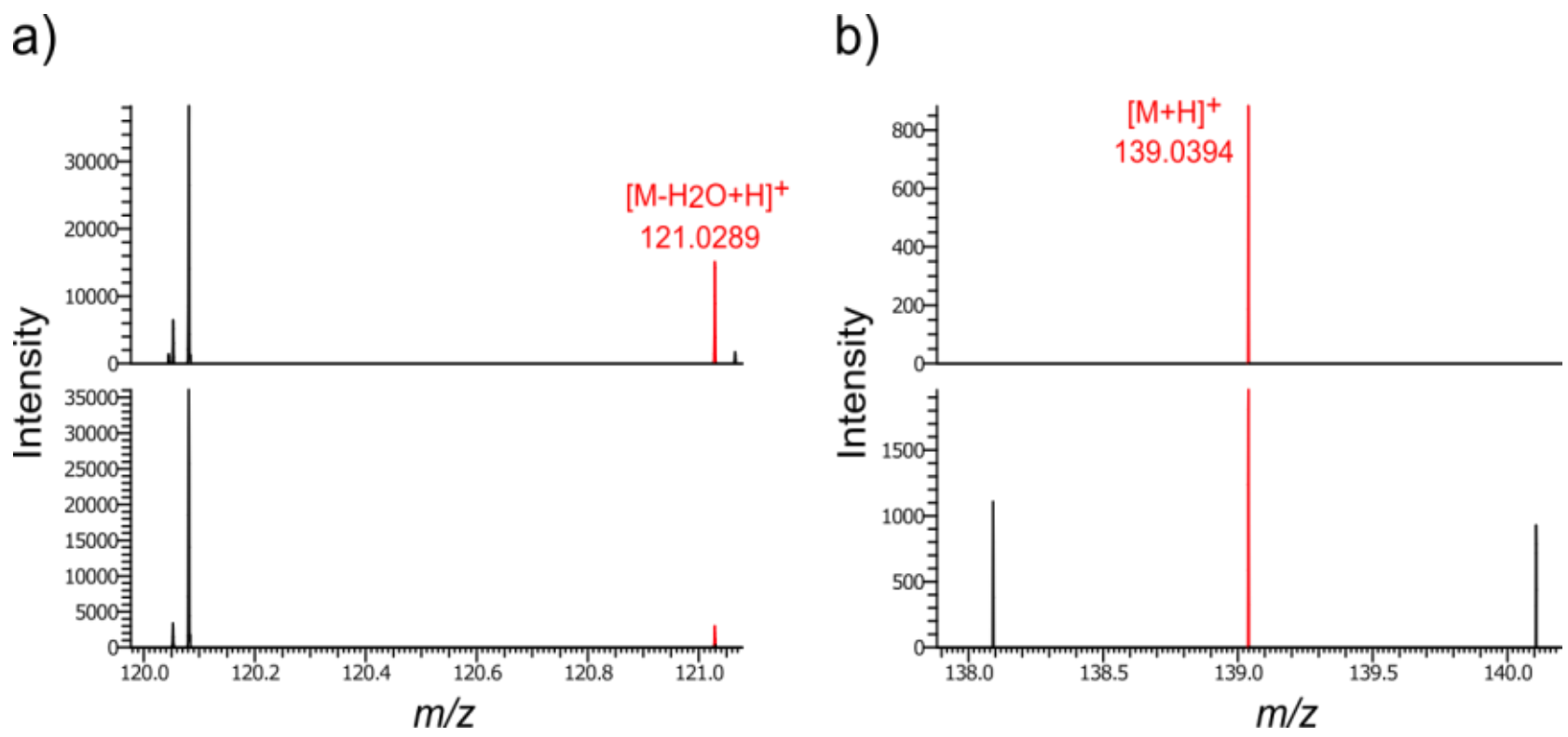

Figure S8: MALDI-MS spectra acquired from the dried-droplet of 4-hydroxybenzoic acid standard analysis (top position) and brain section imaging (bottom position) using the ISM CHCA+DEA: (a) zoomed in at the peak *m/z* 121.0289 ([M-H_2O+H]$^+$) and (b) zoomed in at the peak *m/z* 139.0394 ([M+H]$^+$)

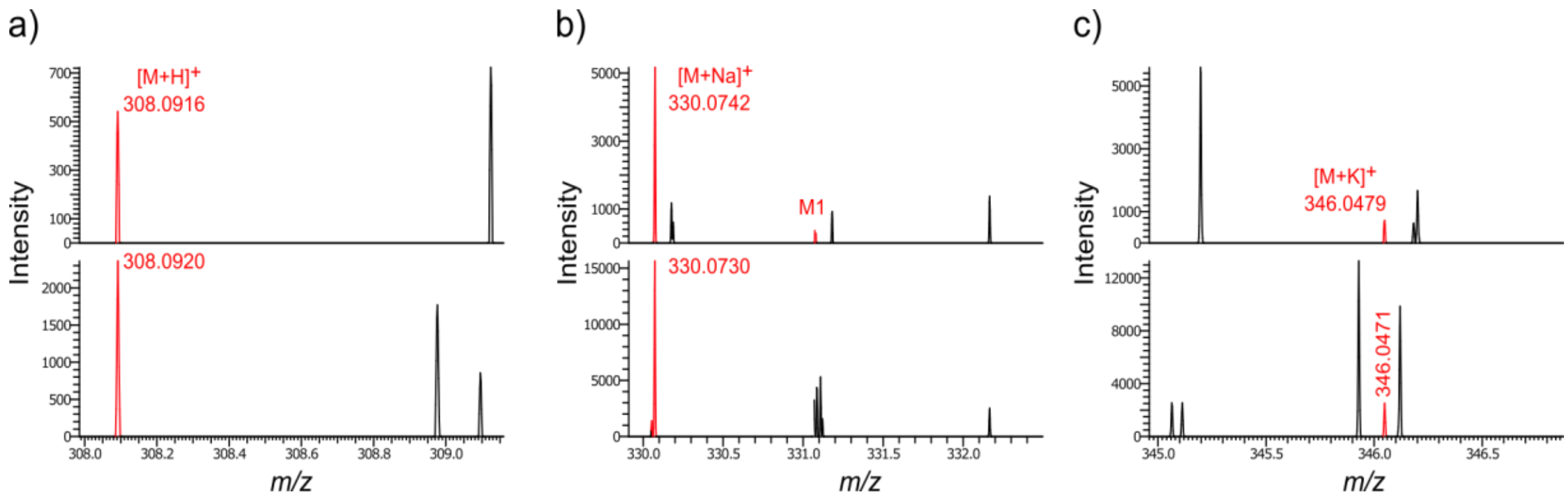

Figure S9: MALDI-MS spectra acquired from the dried-droplet of glutathione standard analysis (top position) and brain section imaging (bottom position) using the ISM CHCA+DEA: (a) zoomed in at the peak *m/z* 308.09 ($[M+H]^+$); (b) zoomed in at the peak *m/z* 330.0742 ($[M+Na]^+$); and (c) zoomed in at the peak *m/z* 346.05 ($[M+K]^+$).

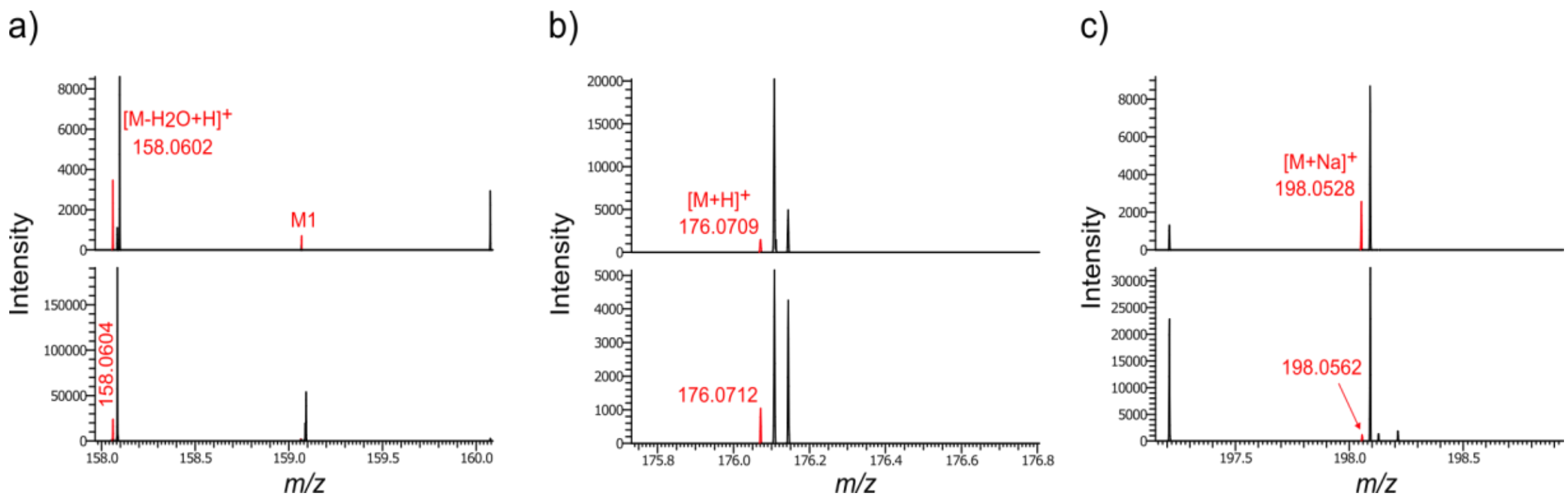

Figure S10: MALDI-MS spectra acquired from the dried-droplet of indoleacetic acid standard analysis (top position) and brain section imaging (bottom position) using the ISM CHCA+DEA: (a) zoomed in at the peak *m/z* 158.06 ($[M-H_2O+H]^+$); (b) zoomed in at the peak *m/z* 176.07 ($[M+H]^+$); and (c) zoomed in at the peak *m/z* 198.05 ($[M+Na]^+$).

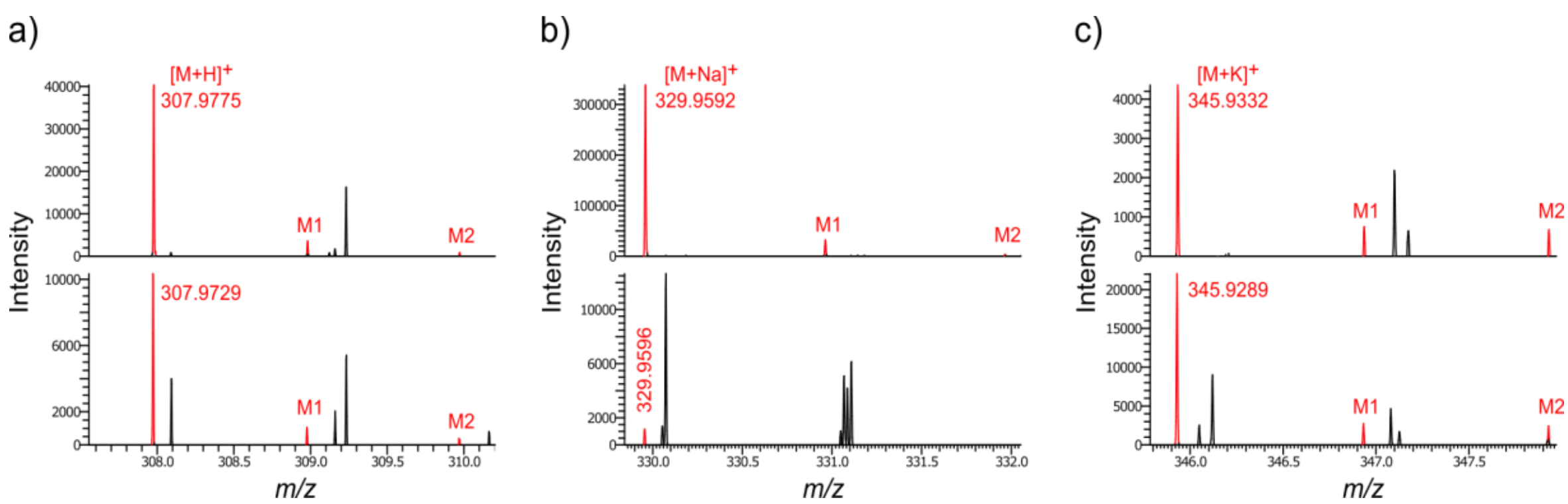

Figure S11: MALDI-MS spectra acquired from the dried-droplet of 3-iodotyrosine standard analysis (top position) and brain section imaging (bottom position) using the ISM CHCA+DEA: (a) zoomed in at the peak *m/z* 307.09 ($[M+H]^+$); (b) zoomed in at the peak *m/z* 329.96 ($[M+Na]^+$); and (c) zoomed in at the peak *m/z* 345.93 ($[M+K]^+$).

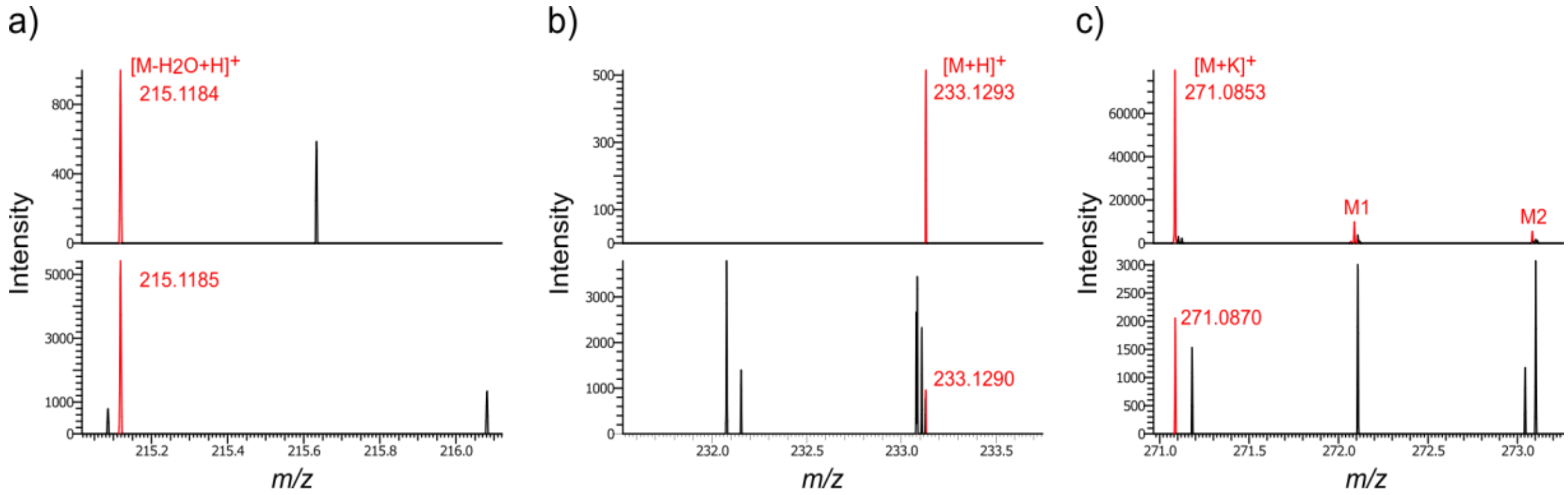

Figure S12: MALDI-MS spectra acquired from the dried-droplet of melatonin standard analysis (top position) and brain section imaging (bottom position) using the ISM CHCA+DEA: (a) zoomed in at the peak *m/z* 215.12 ([M-H_2O+H]$^+$); (b) zoomed in at the peak *m/z* 233.13 ([M+Na]$^+$); and (c) zoomed in at the peak *m/z* 271.08 ([M+K]$^+$).

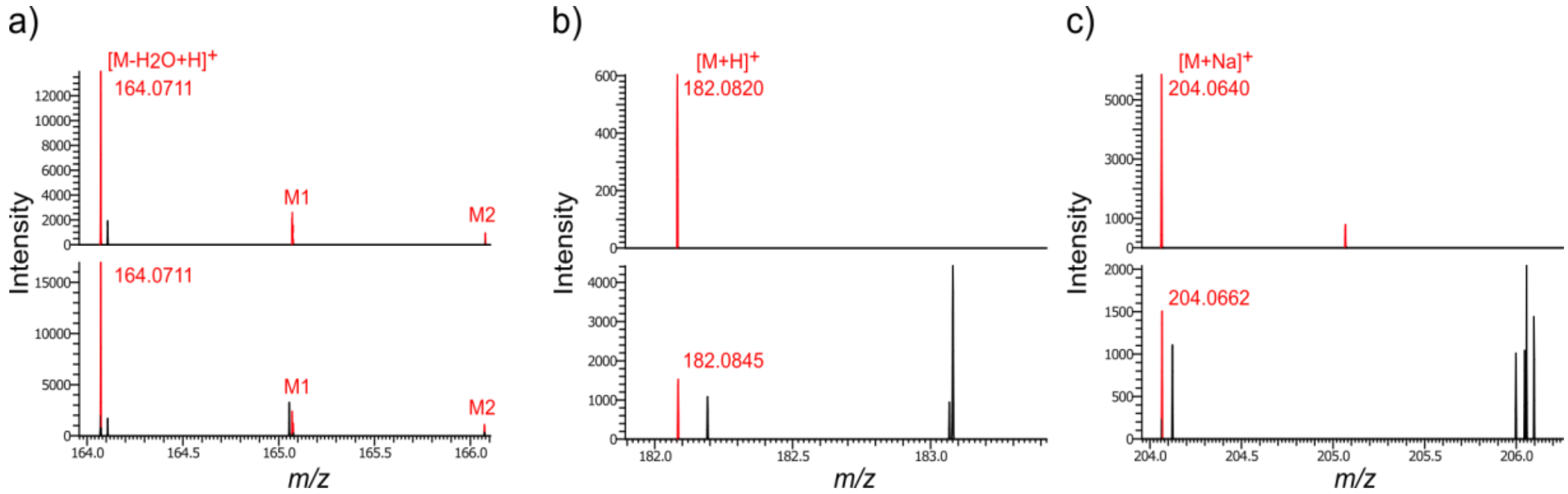

Figure S13: MALDI-MS spectra acquired from the dried-droplet of tyrosine standard analysis (top position) and brain section imaging (bottom position) using the ISM CHCA+DEA: (a) zoomed in at the peak *m/z* 164.07 ([M-H_2O+H]$^+$); (b) zoomed in at the peak *m/z* 182.08 ([M+H]$^+$); and (c) zoomed in at the peak *m/z* 204.06 ([M+Na]$^+$).

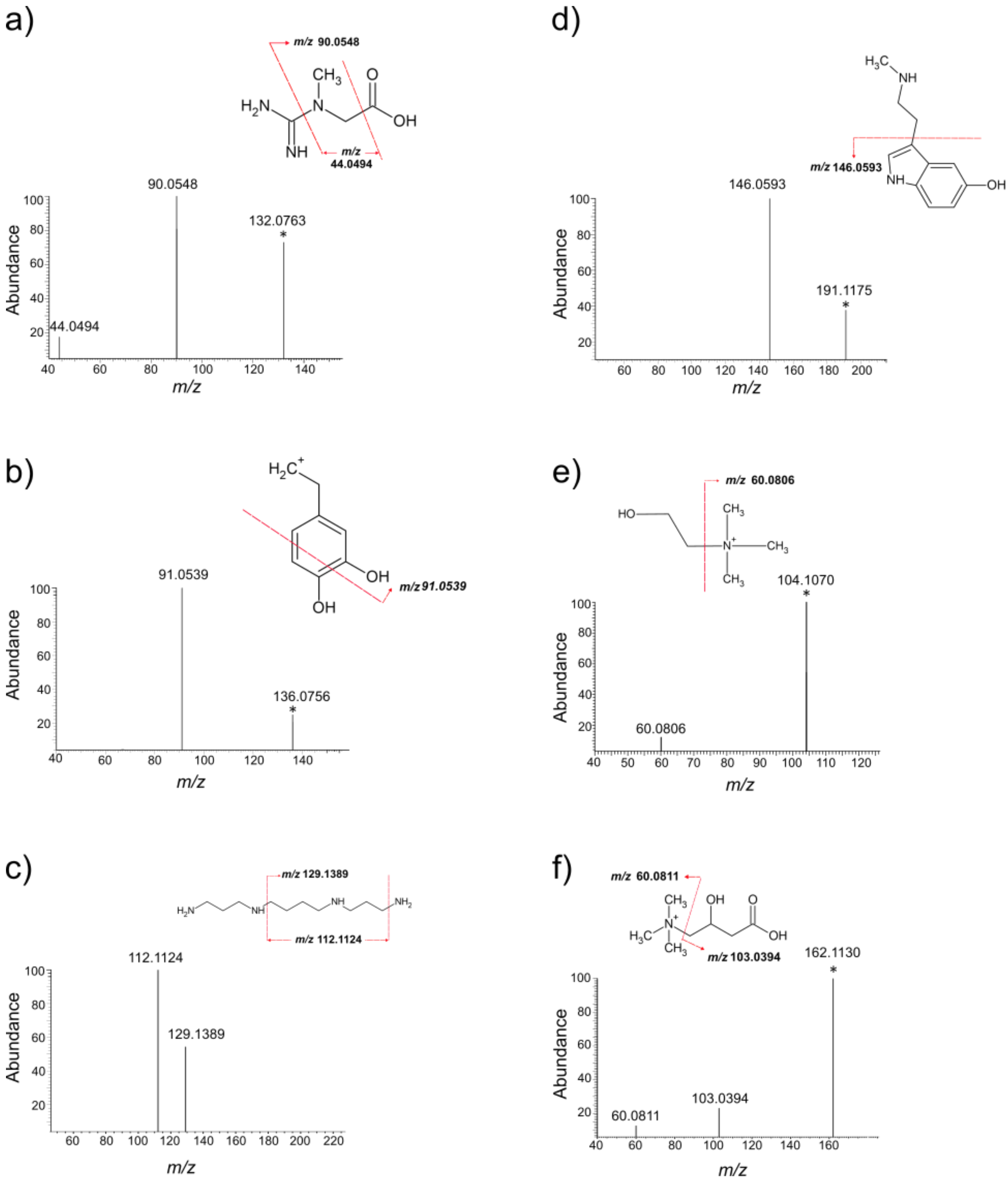

Figure S14: MALDI-MS/MS spectra acquired from the brain section imaging using the ISM CHCA+DEA, with the proposed fragmentation pathways of: (a) creatine; (b) dopamine; (c) spermine; (d) n-methylserotonin; (e) choline; and (f) carnitine.

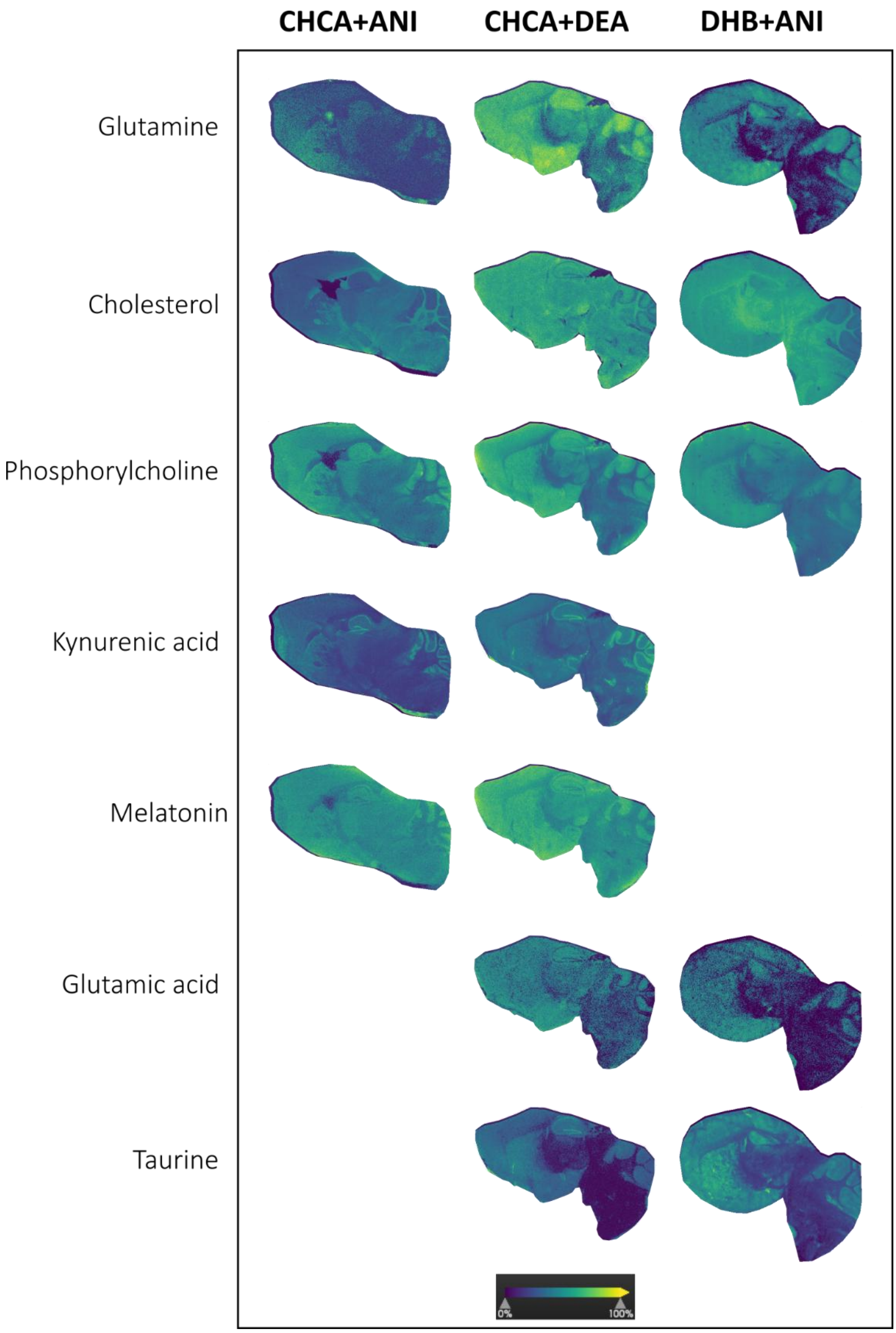

Figure S15: MALDI images of selected metabolites in a mouse sagittal brain section, acquired at 20 µm spatial resolution using the ISMs CHCA+ANI, CHCA+DEA, and DHB+ANI.

Table S2: List of LMWCs (*m/z* < 500 Da) annotated from MALDI-MSI of mouse brain using the ISM CHCA+DEA.

**Compound**	***m/z***	**ppm error (+/-)**	**Adduct type**
Acetaminophen	134.0605	0.04	$[M-H_2O+H]^+$
Acetic acid	136.9403	1.25	$[M+2K-H]^+$
N-Acetyl-L-aspartic acid	251.968	1.76	$[M+2K-H]^+$
L-Acetylcarnitine *	204.1231	0.52	$[M+H]^+$
Acetylcholine	146.1175	4.05	$[M+H]^+$
7-hydroxytrideca-9,11-dienoic acid	303.0774	5	$[M+2K-H]^+$
N-Acetyl-L-glutamate 5-semialdehyde	212.0326	2.9	$[M+K]^+$
cAMP	330.0575	6.0	$[M+H]^+$
Alanine	90.0552	0.02	$[M+H]^+$
L-Alloisoleucine	114.0917	1.55	$[M-H_2O+H]^+$
Arginine *	175.1196	0.6	$[M+H]^+$
L-Aspartic acid	134.0452	0.44	$[M+H]^+$
L-carnitine *	162.1130	3.0	$[M+H]^+$
Cholesterol *	369.3529	2.31	$[M-H_2O+H]^+$
Choline *	104.1071	1.99	$[M+H]^+$
Creatine *	132.0772	0.38	$[M+H]^+$
	114.0665	1.45	$[M-H_2O+H]^+$
L-Cystathionine	223.0762	4.54	$[M+H]^+$
7-Dehydrocholesterol *	367.3371	1.9	$[M-H_2O+H]^+$
Diacetyl *	162.9559	0.89	$[M+2K-H]^+$
Dihydroxyphenyl-Acetaldehyde (DOPAL)	135.0445	0.71	$[M-H_2O+H]^+$

Dihydroxyphenylglycol (DOPEG)	105.0702	1.7	$[M-H_2O+H]^+$
Docosahexaenoic acid	367.2045	1.74	[M+K]+
Docosanedioic acid	409.2740	3.3	$[M+Na]^+$
DOPA	180.0662	1.1	$[M-H_2O+H]^+$
Dopamine	136.0762	0.18	$[M-H_2O+H]^+$
Erythronic acid	137.0462	5.0	$[M+H]^+$
gamma-Aminobutyric acid (GABA)	104.0709	1.99	$[M+H]^+$
	179.9826	1.34	$[M+2K-H]^+$
Ethanolamine	62.0602	4.67	$[M+H]^+$
Glutamic acid *	130.0503	0.64	$[M-H_2O+H]^+$
Glutamine	185.033	0.81	$[M+K]^+$
	191.0409	1.36	$[M+2Na-H]^+$
Glutathione	308.0929	4.33	$[M+H]^+$
	330.0742	4.66	$[M+Na]^+$
	346.0489	4.05	$[M+K]^+$
Hippuric acid	162.0555	0.54	$[M-H_2O+H]^+$
4-Hydroxybenzoic acid (4-HB)	121.0288	0.92	$[M-H_2O+H]^+$
	139.0395	0.09	$[M+H]^+$
Hydroxycholesterol *	385.3478	2.01	$[M-H_2O+H]^+$
5-Hydroxyindoleacetic acid	192.0662	0.94	$[M+H]^+$
	174.0555	0.01	$[M-H_2O+H]^+$
6-Hydroxymelatonin	231.1135	0.98	$[M-H_2O+H]^+$
Indoleacetaldehyde	160.0763	4.52	$[M+H]^+$

5-Hydroxytryptophol	118.0464	0.41	[M-H_2O+H]$^+$
Indole	158.0606	0.01	[M+H]$^+$
Indoleacetic acid	176.0712	0.61	[M-H_2O+H]$^+$
	198.0528	0.51	[M+H]$^+$
	307.9775	1.42	[M+Na]$^+$
Iodotyrosine	329.9592	0.95	[M+H]$^+$
	345.9332	1.78	[M+Na]$^+$
	401.3428	0.95	[M+K]$^+$
7-Ketocholesterol	383.3313	2.34	[M+H]$^+$
	265.9626	0.09	[M-H_2O+H]$^+$
Kynurenic acid	376.1394	1.69	[M+2K-H]$^+$
Kyotorphin	166.9506	2.08	[M+K]$^+$
Lactic acid	215.1184	0.09	[M+2K-H]$^+$
Melatonin	233.1292	1.05	[M-H_2O+H]$^+$
	271.0853	1.05	[M+H]$^+$
	174.0919	1.15	[M+K]$^+$
5-Methoxytryptophol	150.0919	0.5	[M-H_2O+H]$^+$
3-Methoxytyramine	74.0715	0.22	[M-H_2O+H]$^+$
Methylguanidine	123.0557	4.17	[M+H]$^+$
Methylimidazoleacetic acid	143.1184	1.01	[M-H_2O+H]$^+$
N(6)-Methyllysine	176.1076	0.05	[M-H_2O+H]$^+$
(S)-N-Methylsalsolinol	191.1175	0.43	[M-H_2O+H]$^+$
N-Methylserotonin	395.2585	0.95	[M+H]$^+$

MG 18:1	290.0823	2.7	$[M+K]^+$
O-Phosphoethanolamine	124.0162	2.00	$[M-H_2O+H]^+$
	217.9385	1.85	$[M+2K-H]^+$
Palmitoylethanolamide *	282.2802	1.98	$[M-H_2O+H]^+$
4-Methylbenzoic acid	137.0601	3.0	$[M+K]^+$
Phenylacetic acid	119.0495	1.07	$[M-H_2O+H]^+$
Phosphodimethylethanolamine	245.9699	1.08	$[M+2K-H]^+$
Phosphorylcholine *	184.0733	1.85	$[M+H]^+$
Pipecolic acid	154.0267	0.38	$[M+2K-H]^+$
Propionylcarnitine *	240.1221	5.0	$[M+Na]^+$
Propionylcholine	198.0915	4.16	$[M+K]^+$
Pyridoxal	150.0554	1.17	$[M-H_2O+H]^+$
Pyroglutamic acid	112.0397	2.0	$[M-H_2O+H]^+$
Spermine *	203.2237	1.35	$[M+H]^+$
Taurine	163.9784	4.04	$[M+K]^+$
	201.9344	0.75	$[M+2K-H]^+$
Trimethyl-L-lysine	171.1498	1.16	$[M-H_2O+H]^+$
Tyrosine	164.0712	0.7	$[M-H_2O+H]^+$
	182.0820	4.59	$[M+H]^+$

* Compounds detected using the conventional matrix (CHCA or DHB).

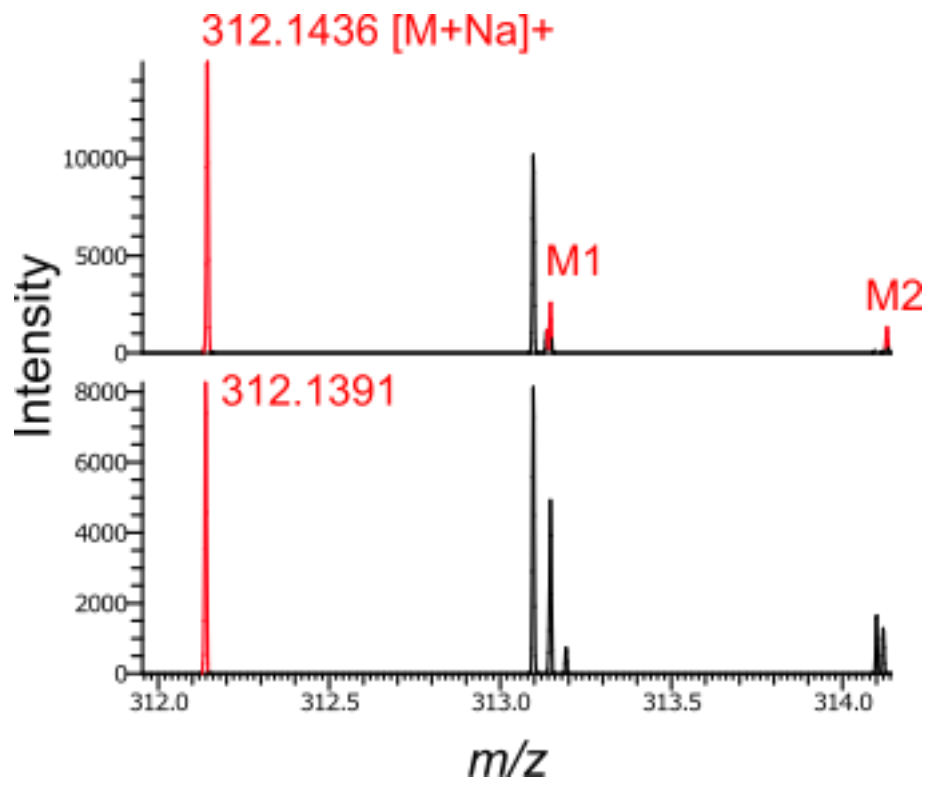

Figure S16: MALDI-MS spectra acquired from the dried-droplet of 3-methyl-glutarylcarnitine standard analysis (top position) and mouse pancreatic section imaging (bottom position) using the ISM CHCA+DEA.

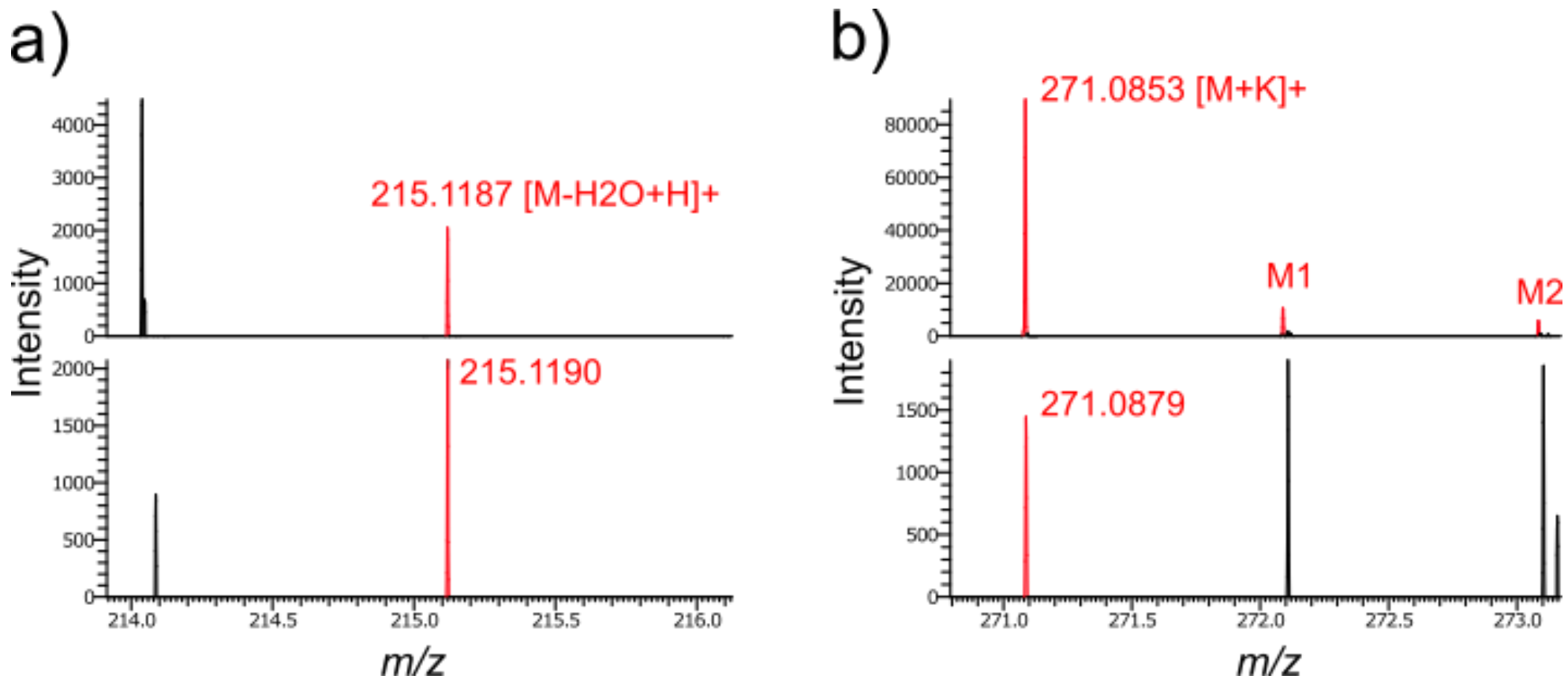

Figure S17: MALDI-MS spectra acquired from the dried-droplet of melatonin standard analysis (top position) and mouse pancreatic section imaging (bottom position) using the ISM CHCA+DEA: (a) zoomed in at the peak *m/z* 215.12 ($[M-H_2O+H]^+$) and (b) zoomed in at the peak *m/z m/z* 271.08 ($[M+K]^+$).

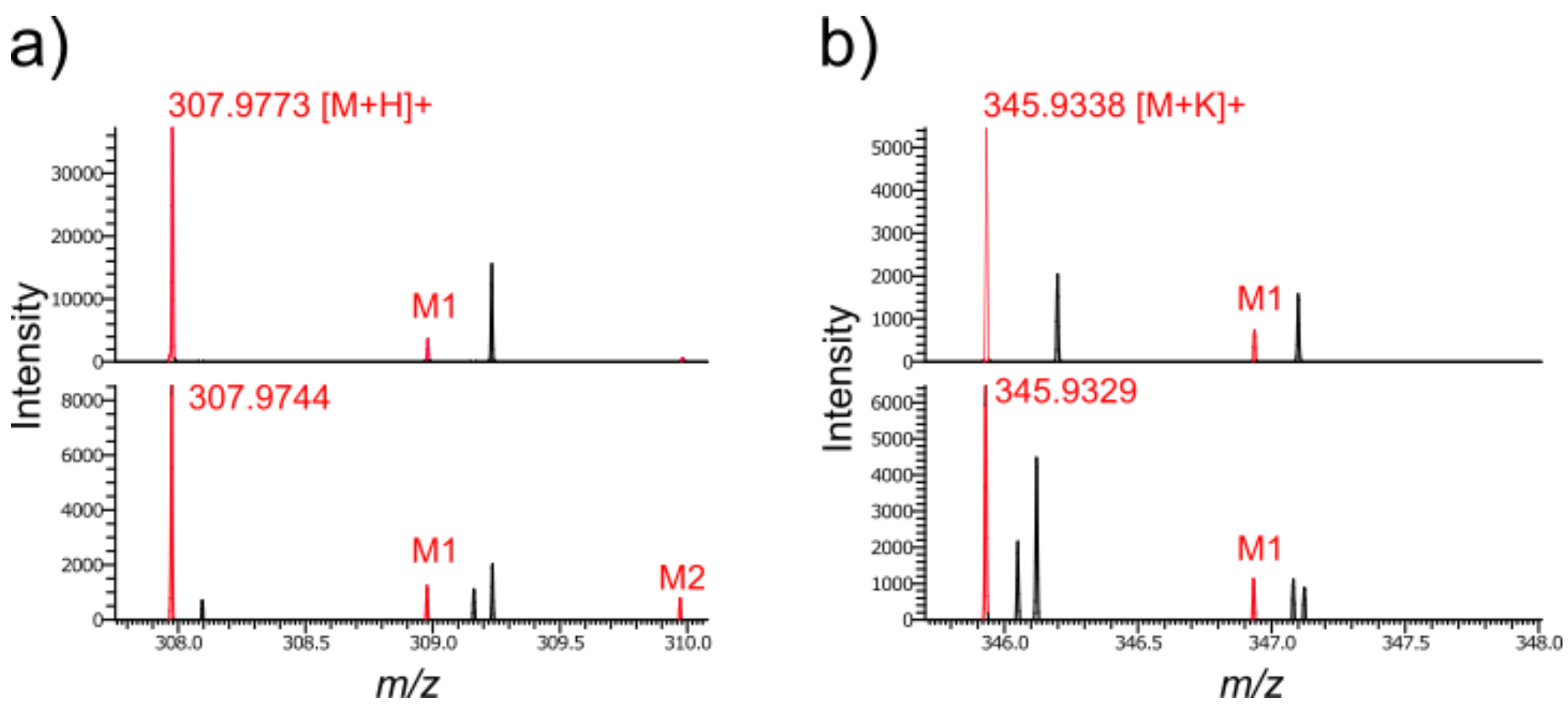

Figure S18: MALDI-MS spectra acquired from the dried-droplet of 3-iodotyrosine standard analysis (top position) and mouse pancreatic section imaging (bottom position) using the ISM CHCA+DEA: (a) zoomed in at the peak *m/z* 307.09 ($[M+H]^+$) and (b) zoomed in at the peak *m/z* 345.93 ($[M+K]^+$).

.

Table S3: List of low-molecular-weight compounds (LMWCs; *m/z* < 500) putatively annotated within the pancreatic islets of Langerhans using the ISM CHCA+DEA.

m/z	**Adduct type**	**ppm error (+/-)**	**Compound /class**
428.1916	$[M-H_2O+H]^+$	5.0	PC 12:4
312.1413	$[M+Na]^+$	1.2	Methylglutarylcarnitine
249.1274	$[M+K]^+$	3.5	Methyl (2E,6Z)-dodecadienoate
237.1426	$[M+Na]^+$	4.9	Oxo-dodecanoic-acid
355.1067	$[M+K]^+$	5.0	FA 16:3
496.3413	$[M+H]^+$	3.3	LPC 16:0
104.1077	$[M+H]^+$	1.9	Choline
307.9779	$[M+H]^+$	0.5	Iodotyrosine
417.2434	$[M-H_2O+H]^+$	3.4	LPA 18:2
215.1185	$[M-H_2O+H]^+$	0.4	Melatonin
417.1726	$[M+K]^+$	3.2	6-beta-hydrocortisol
297.1266	$[M+H]^+$	0.6	Phenylalanyl-methionine
325.1476	$[M+Na]^+$	2.0	Neuromedin B (1-3)
296.1225	$[M+H]^+$	3.1	Tyrosyl-asparagine
369.3571	$[M-H_2O+H]^+$	2.3	Cholesterol
